# Rates and Microbial Players of Iron-Driven Anaerobic Oxidation of Methane in Methanic Marine Sediments

**DOI:** 10.3389/fmicb.2019.03041

**Published:** 2020-01-17

**Authors:** David A. Aromokeye, Ajinkya C. Kulkarni, Marcus Elvert, Gunter Wegener, Susann Henkel, Sarah Coffinet, Thilo Eickhorst, Oluwatobi E. Oni, Tim Richter-Heitmann, Annika Schnakenberg, Heidi Taubner, Lea Wunder, Xiuran Yin, Qingzeng Zhu, Kai-Uwe Hinrichs, Sabine Kasten, Michael W. Friedrich

**Affiliations:** ^1^Microbial Ecophysiology Group, Faculty of Biology/Chemistry, University of Bremen, Bremen, Germany; ^2^MARUM – Center for Marine Environmental Sciences, University of Bremen, Bremen, Germany; ^3^International Max Planck Research School of Marine Microbiology, Max Planck Institute for Marine Microbiology, Bremen, Germany; ^4^Faculty of Geosciences, University of Bremen, Bremen, Germany; ^5^Max Planck Institute for Marine Microbiology, Bremen, Germany; ^6^Alfred Wegener Institute Helmholtz Centre for Polar and Marine Research, Bremerhaven, Germany; ^7^Faculty of Biology/Chemistry, University of Bremen, Bremen, Germany

**Keywords:** anaerobic oxidation of methane, iron oxides, marine sediment, anaerobic methanotrophs, radiotracer, microbial community analysis, stable isotope probing, ANME-2a

## Abstract

The flux of methane, a potent greenhouse gas, from the seabed is largely controlled by anaerobic oxidation of methane (AOM) coupled to sulfate reduction (S-AOM) in the sulfate methane transition (SMT). S-AOM is estimated to oxidize 90% of the methane produced in marine sediments and is mediated by a consortium of anaerobic methanotrophic archaea (ANME) and sulfate reducing bacteria. An additional methane sink, i.e., iron oxide coupled AOM (Fe-AOM), has been suggested to be active in the methanic zone of marine sediments. Geochemical signatures below the SMT such as high dissolved iron, low to undetectable sulfate and high methane concentrations, together with the presence of iron oxides are taken as prerequisites for this process. So far, Fe-AOM has neither been proven in marine sediments nor have the governing key microorganisms been identified. Here, using a multidisciplinary approach, we show that Fe-AOM occurs in iron oxide-rich methanic sediments of the Helgoland Mud Area (North Sea). When sulfate reduction was inhibited, different iron oxides facilitated AOM in long-term sediment slurry incubations but manganese oxide did not. Especially magnetite triggered substantial Fe-AOM activity and caused an enrichment of ANME-2a archaea. Methane oxidation rates of 0.095 ± 0.03 nmol cm^–3^ d^–1^ attributable to Fe-AOM were obtained in short-term radiotracer experiments. The decoupling of AOM from sulfate reduction in the methanic zone further corroborated that AOM was iron oxide-driven below the SMT. Thus, our findings prove that Fe-AOM occurs in methanic marine sediments containing mineral-bound ferric iron and is a previously overlooked but likely important component in the global methane budget. This process has the potential to sustain microbial life in the deep biosphere.

## Introduction

In marine sediments globally, methanogenic archaea form large amounts of the potent greenhouse gas methane (Reeburgh, 2007). Because of a rather effective biological filter – the anaerobic oxidation of methane (AOM) – an estimated 90% of this methane is consumed before escaping from the sediment (Hinrichs and Boetius, 2003; Knittel and Boetius, 2009). AOM is commonly mediated by a consortium of anaerobic methane oxidizing archaea (ANME) and sulfate reducing bacteria (Hinrichs et al., 1999; Boetius et al., 2000; Hinrichs and Boetius, 2003; Knittel and Boetius, 2009), which results in the establishment of a sulfate methane transition (SMT) (Iversen and Jørgensen, 1985; Niewöhner et al., 1998; Jørgensen and Kasten, 2006), a reactive layer in which methane diffusing from the subsurface is oxidized with sulfate diffusing downward from the seawater. In addition to sulfate-coupled AOM (S-AOM), the role of other electron acceptors as additional sinks for methane in marine sediments is not fully established. Metal oxides such as those of iron and manganese have been suggested to serve as additional electron acceptors in AOM (Beal et al., 2009) in a number of terrestrial, coastal and marine environments (Sivan et al., 2011; Chang et al., 2012; Wankel et al., 2012; Segarra et al., 2013; Riedinger et al., 2014; Treude et al., 2014; Egger et al., 2015, 2016a,b, 2017; Oni et al., 2015; Ettwig et al., 2016; Rooze et al., 2016; Scheller et al., 2016; Bar-Or et al., 2017; Martinez-Cruz et al., 2017; Tu et al., 2017; Cai et al., 2018). To date, only few highly enriched cultures from freshwater sediments exist, in which *Candidatus* ‘Methanoperedens nitroreducens’ and *Ca.‘*Methanoperedens ferrireducens’ (members of the clade ANME-2d) were shown to couple iron oxide reduction to anaerobic methane oxidation (Fe-AOM) (Ettwig et al., 2016; Cai et al., 2018):

$${\text{CH}}_{4}+8\,\text{Fe}\,{\left(\text{OH}\right)}_{3}+\,15\,{\text{H}}^{+}\,$$

(1) $$\to {\mathrm{HCO}}_{3}^{-}+\,8\,{\text{Fe}}^{2+}+21\,{\text{H}}_{2}\,\text{O}$$

Although Fe-AOM is thermodynamically feasible, especially with highly soluble iron citrate (Ettwig et al., 2016; Scheller et al., 2016), direct proof for the occurrence of the process in iron oxide-rich marine environments remains elusive.

**FIGURE 1:**
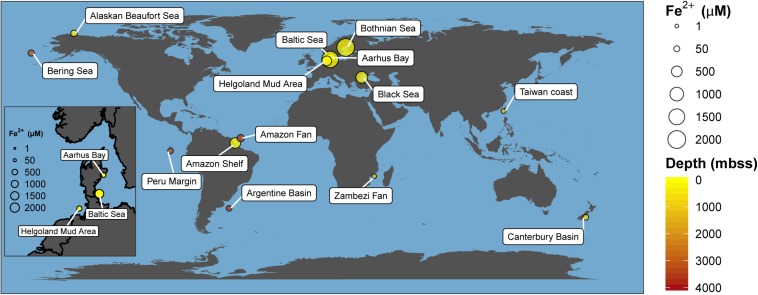
Global map of marine environments with elevated dissolved iron concentrations in the methanic zone. This has been found in both shallow (e.g., Bothnian Sea) and deep-sea environments (e.g., Argentine Basin) of several meters below sea surface (mbss). Presence of elevated dissolved iron concentrations in the methanic zone is currently hypothesized to be primarily driven by Fe-AOM. Absolute dissolved iron concentrations and references are listed in Supplementary Table S1.

Recently, Fe-AOM has been suggested to occur ubiquitously in the methanic zone of iron oxide-rich marine sediments (Riedinger et al., 2014; Egger et al., 2015, 2016a,b, 2017; Oni et al., 2015; Rooze et al., 2016). The existence of an additional methane sink in this zone of continental shelf and margin sediments fueled by iron oxides might hold important implications for biogeochemical cycles of iron and carbon and for microbial life in the energy-limited deep sedimentary biosphere. Elevated concentrations of dissolved iron in pore-water as signatures of ongoing iron reduction, low to undetectable concentrations of sulfate, high contents of buried reactive iron oxides and the presence of methane have been suggested as geochemical signposts for the feasibility of Fe-AOM (Riedinger et al., 2014; Egger et al., 2015, 2016a,b, 2017; Oni et al., 2015; Rooze et al., 2016). Shelf and continental margin sediments have been identified from various parts of the globe as typical depositional environments meeting most of these prerequisites (Figure 1 and Supplementary Table S1). In fact, geochemical modeling suggests Fe-AOM as the likely major mechanism driving iron oxide reduction in the methanic zone of some of these sites (Egger et al., 2015, 2016a,b, 2017; Oni et al., 2015; Rooze et al., 2016). Thus, whether Fe-AOM truly occurs in these environments is discussed controversially. The implication that a second important methane filter exists below the SMT of marine sediments necessitates clarification.

Here, we investigated methanic sediments of the Helgoland Mud Area (HMA) with high sedimentation rates (∼1.6 mm/year – 13 mm/year) and located in the German Bight of the North Sea (Hebbeln et al., 2003), as a representative site bearing the geochemical features of a marine environment where Fe-AOM potentially occurs. In a multi-pronged approach including geochemical analysis of pore-water and sediments, labeling experiments with radiogenic and stable isotopes, molecular biology and lipid stable isotope probing (SIP), we demonstrate the occurrence of Fe-AOM, provide activity rates at near *in situ* conditions and identify ANME-2a as a key microorganism driving this process.

## Results

### Biogeochemical Features of the HMA as a Representative Potential Fe-AOM Site

**FIGURE 2:**
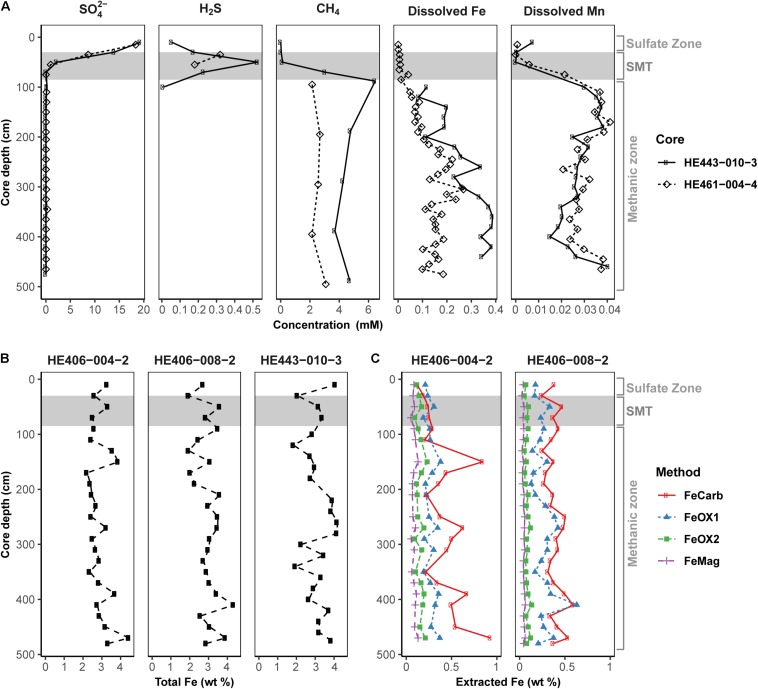
Geochemical profiles reflecting the existence of the geochemical prerequisites for Fe-AOM in the methanic sediments of Helgoland Mud Area. **(A)** Pore-water profiles of sulfate, sulfide, methane, dissolved iron and dissolved manganese in the sediments. **(B)** Solid-phase determination of total Fe contents. **(C)** Distribution of operationally defined iron phases within the sediments (FeCarb: sodium acetate extractable, FeOX1: hydroxylamine-HCl extractable, FeOX2: dithionite extractable and FeMag: oxalate extractable iron oxide phases). Gray area represents the SMT. Sulfate zone, SMT and methanic zones were identified using pore-water profiles in **(A)**.

Pore-water and solid-phase measurements of sediment cores collected during multi-year sampling campaigns to the HMA showed that geochemical preconditions for Fe-AOM occurrence are met in the methanic zone (Figure 2). Pore-water profiles (Figure 2A) consistently showed undetectable sulfate concentrations below the SMT (SMT depth: 30–85 cm; detection limit: 50 μM). Methane concentrations were high (up to 6 mM) below the SMT. Furthermore, dissolved iron concentrations reach up to 380 μM in the methanic zone (>85 cm; Figure 2A). Although elevated dissolved manganese concentrations were also detected suggesting ongoing manganese reduction in the methanic zone, dissolved manganese concentrations were 2–10 folds lower than dissolved iron concentrations (Figure 2A). Solid-phase sediment analysis also revealed that sediments from the methanic zone are replete with metal oxides (mostly iron oxides ranging from 0.49 to 1.64 wt.%, Figures 2B,C; but also, manganese oxides ranging from 0.02 to 0.11 wt.%; Supplementary Figure S1). Therefore, high amounts of buried reactive iron oxides that potentially serve as electron acceptors for AOM in the absence of sulfate are present in the methanic zone of these deposits.

**FIGURE 3:**
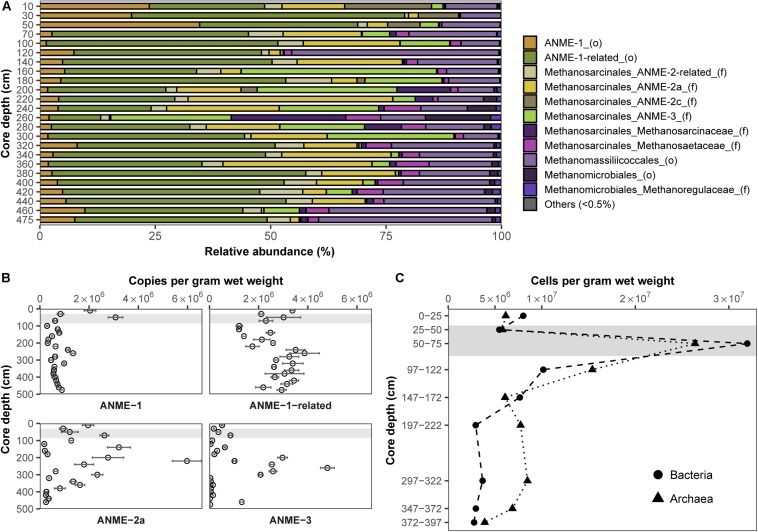
Distribution and abundance of microorganisms in the Helgoland Mud Area sediment core (HE443-010-3). Relative abundances of **(A)** *mcrA* genes and **(B)** *mcrA* gene copies of ANME. Error bars represent 1 s.d. of technical qPCR triplicates. **(C)** Cell counts of potentially active bacteria and archaea based on CARD-FISH. Gray bars within the profiles depict the SMT.

In order to pinpoint microorganisms potentially involved in Fe-AOM at our study site, its microbial community composition was studied at various sediment depths. Based on sequencing of the functional gene marker *mcrA* encoding the methyl coenzyme M reductase alpha subunit (Hales et al., 1996; Luton et al., 2002), we detected phylogenetically diverse ANME populations in sediments from the methanic zone (Figure 3A). An “ANME-1-related” clade (Takeuchi et al., 2011) dominated the methane metabolizing microbial community (up to 55% of *mcrA* genes; Figure 3A). Moreover, estimates of absolute *mcrA* gene copy numbers of the different ANME phylotypes showed that ANME-2a (Boetius et al., 2000; Orphan et al., 2001) and ANME-3 (Niemann et al., 2006) archaea, previously identified as key players during S-AOM, were abundant in the sediments from the iron oxide–rich methanic zone (Figure 3B). Of these groups, ANME-2a was the most abundant, particularly at 220 cm depth (6.0 × 10^6^ copies per gram wet weight; Figure 3B) and was also dominant based on *mcrA* gene sequencing (44%; Figure 3A). Furthermore, the distribution profile of *mcrA* gene copies of the ANME-1-related clade correlates strongly with the dissolved iron concentration across all depths (Pearson’s *r* = 0.64, 95% CI 0.23–0.86, *p* < 0.01; Figures 2A, 3B and Supplementary Table S2). Domain-specific cell counts based on catalyzed reporter deposition fluorescence *in situ* hybridization (CARD-FISH) revealed potentially active archaeal cells in the methanic zone (at least 3.9 × 10^6^ cells per gram wet weight; Figure 3C).

In summary, both pore-water and solid-phase analyses of sediments from HMA show features representative of a sulfate-depleted marine system with potential for iron oxide-driven methane consumption. In addition, these methanic sediments harbor several abundant ANME groups known from S-AOM (Boetius et al., 2000; Orphan et al., 2002; Niemann et al., 2006; Takeuchi et al., 2011) that are potentially involved in Fe-AOM *in situ.*

### Incubation Experiments Demonstrate Iron Oxide-Driven Methane Consumption

**FIGURE 4:**
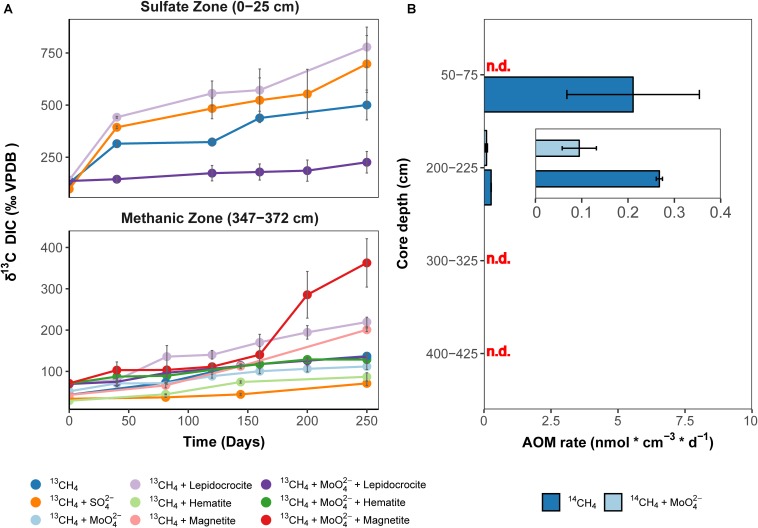
Direct indication for ^13^CH_4_ and ^14^CH_4_ turnover to CO_2_ in incubations with Helgoland Mud Area sediments. **(A)** Change in δ^13^C-DIC values over 250 days in slurry incubation experiments with sediments from the sulfate zone (0–25 cm) and the methanic zone (347–372 cm) with ^13^CH_4_ tracer, *n* = 3, error bars represent 1 s.d. of biological replicates. The ^13^C-increase in the DIC pool serves as proxy for AOM. DIC isotope values in control incubations are provided in Supplementary Figure S11. **(B)** Rates of methane turnover based on ^14^CH_4_ in samples from the SMT (50–75 cm), *n* = 3, and the methanic zone (200–225 cm, 300–325 cm, 400–425 cm), *n* = 2, error bar represents 1 s.d. of biological replicates. INSET: scale adjusted activity rates in the methanic zone. Using ^14^CH_4_, rates of methane turnover were below abiotic control samples in ^14^CH_4_ and molybdate treatment from the SMT and in the methanic zone samples from depths 300–325 cm and 400–425 cm after 8 days. “n.d.”: rates not detected above abiotic controls.

Isotope tracer experiments with ^13^CH_4_ were performed to stimulate iron oxide-driven methane consumption aiming for long-term enrichments, which would provide insights into the ecophysiology of Fe-AOM in marine sediments. The crystalline iron oxides lepidocrocite, hematite and magnetite were shown to be quantitatively important as potential Fe-AOM electron acceptors (Oni et al., 2015) (Figures 2B,C). Therefore, we set up various enrichments with aforementioned iron oxides to identify those iron oxides that are preferentially utilized by Fe-AOM mediating microorganisms in the methanic zone and compared these results with enrichments from the sulfate zone (Figure 4A). Because minimal sulfate concentrations (ranging from 70 to 100 μM) were shown to stimulate S-AOM (Segarra et al., 2015; Timmers et al., 2016), molybdate, a known (Oremland and Taylor, 1978; Oremland and Capone, 1988) and frequently used (Treude et al., 2014; Scheller et al., 2016; Timmers et al., 2016; Bar-Or et al., 2017; Martinez-Cruz et al., 2017) inhibitor of sulfate reduction, was added to a set of replicate incubations. Over 250 days, δ^13^C-DIC values, serving as proxy for methane oxidation, increased continuously in sediment incubations from both, sulfate and methanic zones (Figure 4A). In the methanic zone, incubations amended with iron oxides, in particular magnetite, under inhibition of sulfate reduction, resulted in higher rates of methane oxidation compared to the control incubation amended with ^13^CH_4_ and molybdate (Figure 4A). These observations provided direct indication that sulfate-independent and likely iron oxide-driven AOM occured in the incubations. In contrast, excess sulfate amendment (30 mM) resulted in lower rates of methane oxidation. In sulfate zone sediment incubations, AOM rates were higher compared to the methanic zone incubations, albeit sulfate-driven (Figure 4A). However, amendment with lepidocrocite and molybdate inhibited AOM in the sulfate zone incubations (Figure 4A). Besides Fe-AOM, the potential for manganese oxides to facilitate AOM in the methanic zone was also tested over 250 days. In contrast, amendments with the manganese oxide birnessite did not support AOM activity (Supplementary Figure S2).

As a complementary experiment, we measured AOM activity directly in short-term ^14^CH_4_ incubation experiments at near *in situ* temperatures of 10°C (Oehler et al., 2015) using sediments from the methanic zone and the SMT (see Materials and Methods). In these incubations, methane consumption was observed during the 8-day incubation period at 2 m below sea floor but not at lower depths tested (Figure 4B). Methane was oxidized at a rate of 0.27 ± 0.01 nmol cm^–3^ d^–1^ (see insert, Figure 4B) in the control treatment. For comparison, in SMT sediment incubations, the methane oxidation rate (5.6 ± 2.5 nmol cm^–3^ d^–1^) was 14–31 times higher (Figure 4B). More importantly, in the incubations with methanic sediments, methane oxidation (0.095 ± 0.03 nmol cm^–3^ d^–1^) was detected even under inhibition of sulfate reduction with molybdate (5 mM), indicating a decoupling of AOM from sulfate reduction below the SMT (Figure 4B). Both ^13^CH_4_ and ^14^CH_4_ incubation experiments revealed that iron oxide-driven methane turnover indeed occurs in methanic zone sediments of the HMA.

### Microbial Key Players Involved in Fe-AOM

**FIGURE 5:**
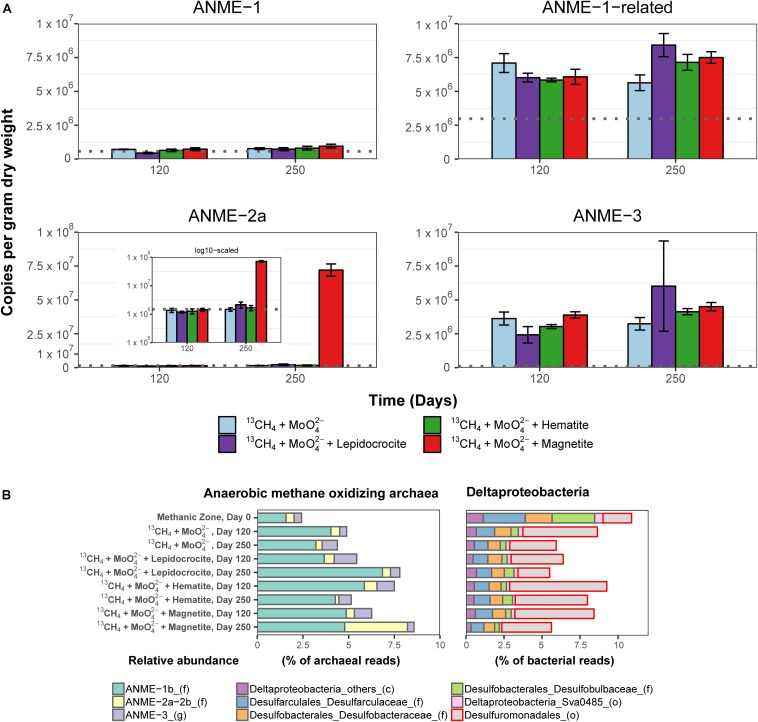
Molecular fingerprints providing insights into microbial activity during ^13^CH_4_ oxidation and potential key players involved in Fe-AOM. **(A)** Abundance of *mcrA* gene copies assigned to different ANME clades in the methanic zone after 120 and 250 days of incubation in molybdate amended incubations from the methanic zone. Gray line in each plot represents estimates of gene copies of the different ANME phylotypes at respective incubation depths (see Figure 3B) as indication for increasing gene copies during the 250-day incubation experiment. INSET: log scaled, adjusted *mcrA* gene copies of ANME-2a. *mcrA* gene copies across all incubations from the sulfate zone and methanic zone are provided in Supplementary Figure S5. Error bars represent 1 s.d. of technical qPCR replicates. **(B)** Relative abundances of ANME and Deltaproteobacteria based on 16S rRNA gene sequencing in the Fe-AOM incubations from the methanic zone after 120 and 250 days. Relative abundance based on total sum scaling of archaeal and bacterial 16S rRNA genes is provided in Supplementary Figures S3, S4, S7, S9.

Potential key players for Fe-AOM in the long-term ^13^CH_4_ incubation experiments were identified by 16S rRNA gene sequencing, *mcrA* gene qPCR of specific ANME phylotypes, lipid SIP of bacterial fatty acids and archaeal ethers, as well as *pmoA* gene amplification and cloning. In incubations of methanic sediments showing Fe-AOM, 16S rRNA gene sequences of detected archaeal methane-oxidizers were affiliated to ANME-1b, ANME-2a/2b, ANME-3 (up to 8.5% of all archaeal sequences; Figure 5B) but not ANME-2c/2d (Supplementary Figures S3, S4). More importantly, in magnetite-molybdate incubations, ANME-2a/2b increased strongly in relative 16S rRNA gene sequence abundance (7–40% of total ANMEs) and ANME-2a specific *mcrA* gene copies (50-fold, Figure 5A) between 120 and 250 days. Thus, in concert with the highest δ^13^C-DIC recorded (Figure 4A), ANME-2a seems to perform Fe-AOM under inhibition of sulfate reduction in magnetite added incubations of sediment from the methanic zone. Moreover, ANME-1-related and ANME-3 were stimulated as well in the other Fe-AOM incubations (Figures 4, 5A). However, a similar trend was observed without molybdate addition and in N_2_ amended controls (Supplementary Figures S3–S5). The stimulation of ANMEs in the N_2_ controls might have been due to methane supply via co-occurring methanogenesis in these incubations (Supplementary Figure S6) recently termed “cryptic methane cycling” and detected in the SMT of Aarhus Bay and other marine sediments (Beulig et al., 2018; Maltby et al., 2018; Xiao et al., 2018).

Known dissimilatory iron reducers from the order Desulfuromonadales were present in all ^13^CH_4_ amended incubations (up to 6.4%, Figure 5B and Supplementary Figure S7). Their increase (ca. 5%) in relative 16S rRNA gene sequence abundance over 250 days is in tandem with the observed iron reduction detected by measuring dissolved iron over time (Supplementary Figure S8), and thus, they might serve as potential iron oxide-reducing partner bacteria in AOM (Chang et al., 2012; Tu et al., 2017). Besides the Desulfuromonadales, unclassified Gammaproteobacteria were highly stimulated compared to day 0 across all incubations in both geochemical zones (Supplementary Figure S9). Of these, known aerobic methanotrophs were below 0.2% of total bacteria 16S rRNA genes and so far nothing is known regarding members of Gammaproteobacteria being partners of ANME.

**FIGURE 6:**
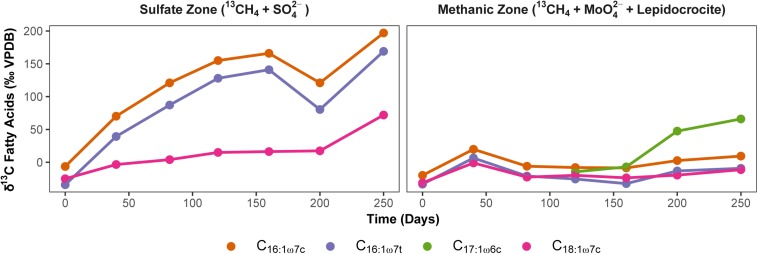
Uptake of ^13^CH_4_ label into bacterial polar lipid fatty acids during AOM. Development of carbon isotopic composition of dominant bacterial fatty acids (‰ VPDB) over time during S-AOM in the sulfate zone (supplemented with sulfate) and Fe-AOM in the methanic zone (supplemented with molybdate and lepidocrocite) is presented. Complete list of δ^13^C values of fatty acids and total uptake by each fatty acid is given in Supplementary Tables S3, S4.

In order to exclude the possible involvement of methanotrophic bacteria in Fe-AOM (Bar-Or et al., 2017; Martinez-Cruz et al., 2017), we studied ^13^C uptake from ^13^CH_4_ into bacterial lipids during Fe-AOM and for comparison, S-AOM. Minor label incorporation into bacterial lipids could be observed in both set-ups (Figure 6 and Supplementary Tables S3, S4) with a maximum incorporation of +60 and +200‰ under Fe-AOM and S-AOM, respectively, after 250 days. We also checked for the presence of *pmoA* genes, a molecular marker for methanotrophic bacteria (Murrell et al., 1998) but amplification was non-specific with the *pmoA* primers used (Supplementary Figure S10). Cloning and sequencing of these products confirmed the non-specific amplification (Supplementary Table S5), and thus, the involvement of methanotrophic bacteria in methane oxidation in our incubations is unlikely, which is corroborated by aerobic methanotrophs being below 0.2% of total bacterial 16S rRNA genes. Investigation of archaeal ether lipids in selected AOM incubations did not show ^13^C-label incorporation during the 250-day incubation from both the methanic and the sulfate zone (Supplementary Table S6).

## Discussion

Anaerobic oxidation of methane in marine sediments is a crucial biological filter that mitigates the flux of the greenhouse gas methane into the ocean and eventually the atmosphere. Previous studies in marine environments extensively documented a coupling of AOM to sulfate reduction in the SMT (Hinrichs et al., 1999; Boetius et al., 2000; Hinrichs and Boetius, 2003; Knittel and Boetius, 2009). Coupling of methane oxidation to nitrate and metal oxide reduction was shown to occur in freshwater environments (Segarra et al., 2013; Ettwig et al., 2016; Cai et al., 2018) where sulfate concentrations are limited but S-AOM predominates as the biological methane filter in marine sediments. Geochemical evidence (Riedinger et al., 2014; Egger et al., 2015) alongside with enrichment efforts replacing sulfate with metal oxides (Beal et al., 2009) and humic acids (Scheller et al., 2016) in sulfate-rich marine sediments hinted toward metal oxide-dependent AOM as additional marine methane sink. However, unlike with S-AOM, a direct environmental signature for metal oxide-dependent AOM is so far lacking. Here, we used a multi-pronged approach to show that iron oxide-driven AOM (Fe-AOM) occurs in the methanic zone of iron oxide-rich marine sediment and especially identified ANME-2a as a key microorganism involved.

### Direct Evidence for Fe-AOM in Methanic Sediments

By showing (I) the turnover of ^14^CH_4_ and ^13^CH_4_ to CO_2_ and (II) the increased gene copy numbers of ANME phylotypes, especially ANME-2a, in incubation experiments stimulating Fe-AOM, our study provides direct evidence for Fe-AOM and identifies ANME as important players during Fe-AOM in the methanic zone of marine sediments. At our study site of HMA, Fe-AOM in the methanic zone occurs at a low rate of 0.095 ± 0.03 nmol cm^–3^ d^–1^, which is roughly ∼2% of that of S-AOM (5.6 ± 2.5 nmol cm^–3^ d^–1^) in the SMT (Figure 4B). Modeled estimates from coastal sediments of the Bothnian Sea also suggested a 3 vs. 97% contribution of Fe-AOM and S-AOM, respectively, to the overall methane consumption (Egger et al., 2015). Similarly, AOM under molybdate inhibition was found in short-term incubations from the methanic zone of Alaskan Beaufort Sea sediment (Treude et al., 2014). However, unlike the Alaskan Beaufort Sea sediment where *in situ* sulfate concentrations (30–500 μM) might still fuel S-AOM (Treude et al., 2014), sulfate concentrations were below detection (<50 μM) in the methanic zone of multiple sediment cores from the HMA (Figure 2A; Oni et al., 2015).

Pore-water profiles show that manganese reduction is also ongoing in the methanic zone of the HMA (Figure 2A), however iron reduction is quantitatively more important (up to 10 folds higher; Figure 2A). In addition, the manganese oxide birnessite suppressed AOM activity (Supplementary Figure S2) in a direct contrast to what was observed with different iron oxides (Figure 4A). Therefore, iron oxides are the more likely electron acceptors for methane oxidation below the SMT in HMA. Previously, Beal et al. (2009) had found a stronger stimulation of AOM in the presence of birnessite than with added ferrihydrite in enrichments with sediment from the SMT of Eel River Basin; however, cryptic sulfur cycling might have occurred in their incubations as sulfate reduction was not inhibited. In addition, microbial communities in HMA and Eel River Basin sediment may differently respond to the presence of manganese oxide. Among the reactive iron minerals present in the methanic zone, magnetite, which quantitatively is the least important iron oxide fraction in the sediments (Figure 2C), stimulated Fe-AOM most strongly, suggesting that this iron oxide mineral could be important for *in situ* Fe-AOM. Similar findings were obtained from incubation experiments with lake sediments (Bar-Or et al., 2017) and supported by a geochemical modeling study on Baltic Sea sediments (Egger et al., 2017). The underlying mechanism for magnetite dependent AOM is unclear from the dataset presented; however, ‘cryptic’ methane cycling is feasible (Beulig et al., 2018, 2019). This process would be fueled by direct interspecies electron transfer (DIET; Kato et al., 2012; Rotaru et al., 2014) with magnetite serving as conductor based on its (semi)conductive properties, facilitating electron transfer to methanogens, which in turn perform CO_2_ dependent methanogenesis in the background. This reasoning is supported by the observation of on-going methanogenesis in the N_2_ control incubations and detection of lower levels of dissolved iron in the magnetite amended incubations over time (Supplementary Figures S6, S8). Bar-Or et al. (2017) recently demonstrated the importance of methanogens as electron outlet in similar incubation set-ups, in which addition of methanogenesis inhibitor 2-bromoethanesulfonate shut down Fe-AOM. In both, ^13^CH_4_ and ^14^CH_4_ slurry incubations, we observed that S-AOM was suppressed in the methanic sediment incubations, while Fe-AOM was suppressed in sulfate-rich sediment incubations (Figure 4). These observations indicate a geochemical niche separation that possibly occurs in the environment with sulfate and ferric iron as electron acceptors for AOM in the sulfate zone and methanic zone, respectively.

Our short-term radiotracer experiments suggest that Fe-AOM rates in the methanic zone are apparently low to undetectable as we only observed Fe-AOM at around ∼2 m below sea floor during our short-term 8-day incubation period (Figure 4B). The undetectable rates in more deeply buried sediments may be due to the decreasing cell numbers of ANME with increasing sediment depth (Figures 3B,C). In strong contrast, the long-term incubations with sediments from ∼3 m below sea floor (Figure 4A) show that Fe-AOM occurs in these deeper sediments given longer incubation time. In the surface sediments of Chowder Hill hydrothermal vents, characterized by a high flux of methane, Fe-AOM rates were an order of magnitude higher than what was observed in this study (Wankel et al., 2012; Table 1), but the sediment depths are shallow and above the SMT (16 cmbsf), unlike the HMA where methanic zone Fe-AOM rates were obtained. Other previously published Fe-AOM rate estimates from marine sediments are either based on ^13^CH_4_ enrichment studies from sulfate-rich sediments or geochemical modeling (Table 1). Therefore, Fe-AOM rates obtained from our short-term incubations represent novel unambiguous experimental demonstration of Fe-AOM from sulfate-depleted subsurface methanic marine sediments. Generally, Fe-AOM rates, not just in marine sediments, are low (Table 1) perhaps owing to the difficulty in accessing iron oxides as an electron acceptor (Lalonde et al., 2012) by microbes involved in the process or as a result of lower cell numbers, typically found with increasing sediment depth in marine sediments (Kallmeyer et al., 2012). But given the estimated global volume of sediments below the SMT (10^8^ km^3^ or 32% of total subsurface) (Bowles et al., 2014), considerable amounts of methane could be consumed over substantial time-scales in the methanic zone before upward diffusion into the SMT. The Fe-AOM rates (Table 1), which were obtained either by geochemical modeling, radiotracer based activity measurements or enrichment studies from both freshwater and marine environments, thus indicate that Fe-AOM is an additional methane sink in ferruginous environments.

**TABLE 1:** Estimated Fe-AOM rates in sediments from various freshwater and marine environments.

**Ecosystem**	**Environment**	**Sediment zone**	**Fe-AOM Rates (μmol CH_4_ cm^–3^ yr^–1^)**	**Data collection source**		**References**
				**Method**	**Fe Oxide**	
Marine	North Sea	Methanic	0.0347	^14^CH_4_ incubations	N.A.	This study
	Eel River Basin seep^§^	Surface	^∗^6	^13^CH_4_ incubations	Ferrihydrite	Beal et al., 2009
	Chowder Hill hydrothermal vent	Surface	59	^14^CH_4_ incubations	N.A.	Wankel et al., 2012
	Black Sea	Methanic	0.00001459	Geochemical modeling estimates	N.A.	Egger et al., 2016a
	Baltic Sea	Methanic	0.0011	Geochemical modeling estimates	N.A.	Egger et al., 2017
	Bothnian Sea	Surface	^∗^1.3	^13^CH_4_ incubations	Ferrihydrite	Egger et al., 2015
	Santa Monica Basin seep^§^	Surface	^∗^292	^13^CH_4_ incubations	Ferric citrate	Scheller et al., 2016
			^∗^36.5	^13^CH_4_ incubations	Ferric EDTA	Scheller et al., 2016
Freshwater	Lake Kinneret	Surface	1.3	^13^CH_4_ incubations	Amorphous Fe(III) oxide	Sivan et al., 2011
	Danish Lake Ørn	Surface	13	^14^CH_4_ incubations	N.A.	Norði et al.,2013
	Dover Bluff salt marsh	Surface	1.4	^14^CH_4_ incubations	Ferrihydrite	Segarra et al., 2013
	Hammersmith Creek river	Surface	4.5	^14^CH_4_ incubations	Ferrihydrite	Segarra et al., 2013

*Process rates were presented as annual rates for comparability between the different environments. The rates presented above are from iron oxide-rich geochemical zones of marine or freshwater sediments unless otherwise stated. ^∗^Rates converted from μmol CO_2_ produced given the assumption that CH_4_ conversion to CO_2_ proceeds in a 1:1 ratio (equation 1). ^§^Represents studies obtained from sulfate-rich geochemical zones. So far, activity based Fe-AOM rates obtained from deep sedimentary biosphere i.e., at 1 m below the seafloor (Jørgensen and Boetius, 2007), is only available from the HMA. Other environments where Fe-AOM was previously demonstrated were therefore referred to as surface sediments in the table above as the sediment depths evaluated are below 1 m. N.A., not available.*

### ANME-2a Identified as Key Player Involved in Fe-AOM

While ANME clades ANME-1, ANME-2 and ANME-3 perform S-AOM (Boetius et al., 2000; Orphan et al., 2001, 2002; Niemann et al., 2006), knowledge on microbial key players involved in Fe-AOM is limited. In the few studies that demonstrated potential for Fe-AOM in marine sediments, either the microbial key players were not shown (Egger et al., 2015) or the geochemical preconditions for Fe-AOM do not exist in the environment where the sediments were obtained (Beal et al., 2009; Scheller et al., 2016). Sequencing of *mcrA* genes from the methanic zone sediments revealed that the canonical ANME-1 were the least dominant ANME group therein (Figure 3A). qPCR analysis of the *mcrA* gene further confirmed that ANME-1, although present, were not involved in Fe-AOM as there was no stimulation of this phylotype based on gene copies over time (Figure 5A). ANME-1-related gene copies correlated positively with the *in situ* dissolved iron concentrations (Supplementary Table S2) but was not the most important group stimulated in our incubation experiments. The general stimulation of ANME-1-related and AMNE-3 both in the AOM performing incubations and the N_2_ controls (Supplementary Figure S5) limits the interpretation that these groups were clearly involved in Fe-AOM. Lack of incorporation of ^13^C-label in archaeal lipids in the methanic zone incubations with lepidocrocite suggested that ANMEs were neither directly assimilating CH_4_, nor indirectly incorporating DIC into their biomass, with the latter previously shown to be the dominant mode for their relatives mediating S-AOM (Kellermann et al., 2012). This is, moreover, in accordance with archaeal lipid isotopes from Fe-AOM incubations using lake sediments where associated methanogens were similarly found with only marginal incorporation of ^13^C-label (Bar-Or et al., 2017).

Gene copies of ANME-2a, however, were strongly enriched between day 120 and 250 in magnetite-molybdate incubation experiments (Figure 5A), which coincided with increased ^13^CH_4_ turnover (Figure 4A). Moreover, ANME-2a were also abundant in the methanic zone (Figure 3B). The enrichment of ANME-2a in magnetite-molybdate amended incubations supported by their high gene copy numbers in the methanic zone indicates that ANME-2a perform Fe-AOM. Therefore, our successful enrichment of ANME-2a with magnetite as electron acceptor advances our knowledge on Fe-AOM substantially as we identify for the first time ANME-2a as a microbial key player for Fe-AOM in marine environments.

In terrestrial mud volcanoes where Fe-AOM is also suggested to occur, high correlation between gene copies of Desulfuromonadales and ANME-2a was taken as indication for ANME-2a to oxidize methane with Desulfuromonadales as iron oxide reducing partners (Chang et al., 2012; Tu et al., 2017). While dissimilatory iron oxide reducers (e.g., Desulfuromonadales) have not been clearly shown to act as partners for ANMEs, the increased relative abundance of Desulfuromonadales 16S rRNA genes (Figure 5B) cannot be ignored. However, whether they are syntrophic partners of ANME in Fe-AOM requires further research as a clear indication for bacteria partner organisms was not obtained from the long-term incubations. Possibly, ANME-2a completely oxidize CH_4_ to CO_2_ without a bacterial partner. A previous study showed that ANME-2a, like ANME-2d, possess a variety of multi-heme cytochromes in their genome (Wang et al., 2014), and based on their metatranscriptome datasets they argued that ANME-2a may exist without bacterial partners in specific marine sediments. Therefore, they also could analogously perform Fe-AOM like their freshwater derived ANME-2d relatives (Ettwig et al., 2016; Cai et al., 2018). Scheller et al. (2016) presented similar arguments after observing that ANME-2a and -2c archaea were able to decouple their syntrophic relationship with sulfate reducing bacteria when provided with artificial electron acceptors. Both studies indicate that ANME-2 archaea respire solid electron acceptors via direct extracellular electron transfer. A similar electron transfer mechanism may appear in our magnetite-molybdate amended incubations. This hypothesis offers a promising pathway to isolate ANME-2 archaea and should be a focus of future studies to glean a better understanding of the ecophysiology of methane cycling archaea.

### Methanotrophic Bacteria Are Not Involved in Marine Sediment Fe-AOM

Based on their biochemistry Gammaproteobacterial methanotrophic bacteria such as members of the genus *Methylobacter* should be strict aerobes (Kalyuzhnaya et al., 2013; Chistoserdova, 2015). Yet recently, based on DNA and lipid stable isotope probing approaches, methylotrophic bacteria were suggested to be involved in Fe-AOM in lake sediments (Bar-Or et al., 2017; Martinez-Cruz et al., 2017). In these studies, direct incorporation of ^13^C-label from CH_4_ into bacterial lipids was shown to be as high as +3200‰ (Bar-Or et al., 2017) when methanotrophic bacteria were directly involved in methane turnover. Bacterial lipid SIP data from our study with significantly lower label incorporation (+60‰, Figure 6) suggest a rather indirect incorporation of ^13^C, most likely from ^13^C-DIC, which is produced via ^13^CH_4_ oxidation but diluted into slurries. Given the substantially lower incorporation of ^13^C-label in bacterial fatty acids (Figure 6 and Supplementary Table S4) compared to lake sediment incubations (Bar-Or et al., 2017; Martinez-Cruz et al., 2017), there was no evidence to support direct uptake of label from CH_4_ into bacteria in samples from both, the sulfate and the methanic zone. Lipid SIP results were corroborated by the lack of detection of methanotrophic bacteria specific *pmoA* genes in Fe-AOM performing incubations (Supplementary Figure S10 and Supplementary Table S5). Thus, in contrast to Fe-AOM in lake sediments, involvement of methanotrophic bacteria was conclusively ruled out in our marine sediment incubations.

### Environmental Significance of Fe-AOM in Marine Sediments

Our study shows that Fe-AOM is an additional sink for methane in subsurface coastal and marine sediments where methane and iron oxides co-exist with low to undetectable sulfate concentrations. These environments are typically characterized by high sedimentation rates and/or high loading of iron oxides, facilitating the burial of reactive iron oxides beneath the SMT. Such environments bearing elevated dissolved iron concentrations as indicator for ongoing iron reduction are widely distributed from shallow sediments on coastal shelves to deep sub-seafloor settings at lower continental margins (Figure 1; Aller et al., 1986; Schulz et al., 1994; Kasten et al., 1998; Hensen et al., 2003; D’ Hondt et al., 2004; März et al., 2008, 2018; Fulthorpe et al., 2011; Holmkvist et al., 2011; Lim et al., 2011; Takahashi et al., 2011; Riedinger et al., 2014; Treude et al., 2014; Egger et al., 2015, 2016a,b, 2017; Oni et al., 2015; Rooze et al., 2016). Besides, Fe-AOM might have been an important methane sink in the early Archean before the accumulation of sulfate in the ocean (Konhauser et al., 2005) and it was previously suggested that Fe-AOM should be considered in methane oxidation estimates from marine environments (Riedinger et al., 2014). Our study provides rate estimates for Fe-AOM and shows that ANME-2a archaea are key players for the process in iron oxide-rich methanic marine sediments. Could Fe-AOM account for a large proportion of AOM in marine sediments? A back-of-the-envelope calculation (Supplementary Table S8) taking only the SMT based diffusive modeled methane flux of inner shelf sediments (0–50 m water depths, Egger et al., 2018) into account, showed that a methanic zone thickness of ∼6 m would be required – which is feasible – albeit there is only our HMA based Fe-AOM measured rate so far. However, the stoichiometry of the reaction – 8 moles of iron(III) per mole of methane fully oxidized to CO_2_ – requires sediments rich in reactive iron oxides. Thus, an evaluation of Fe-AOM on a global scale with rate measurements and the determination of reactive iron-oxide pools is required to advance current diagenetic models (Egger et al., 2015, 2016a,b, 2017; Rooze et al., 2016) and improve our understanding of its contribution to methane budgets in marine environments.

## Materials and Methods

### Experimental Design

This study took a multi-year sampling approach to study Fe-AOM in the HMA sediments. Sediment sampling of 5 m gravity cores was done from the same site over four different years to understand the microbial ecophysiology behind Fe-AOM (see Table 2). In order to ascertain the potential of HMA to harbor Fe-AOM, some gravity cores were sampled on board to conduct geochemical solid-phase (e.g., total Fe and Mn) and pore-water analyses (dissolved iron and manganese, CH_4_, SO_4_^2–^ etc.). Simultaneously, sediment samples were frozen on board and were used for identifying *in situ* microbial populations that may be involved in AOM, using molecular techniques such as next generation sequencing and qPCR. The other gravity cores were transported to the lab at 4°C and used to set up long-term (over 250 days) anaerobic microbial enrichments amended with ^13^CH_4_ and different iron oxides and birnessite (manganese oxide). Fe- and Mn-AOM activity was detected by measuring ^13^C-DIC turnover over regular intervals and enriched archaeal methane oxidizers were identified using 16S rRNA gene sequencing and phylotype specific qPCR. Lipid-SIP was performed to confirm the absence of aerobic methanotrophs. Lastly, using freshly sampled sediments, short term (8 days) incubations amended with ^14^CH_4_ were set up to quantify Fe-AOM rates. Thus, using geochemical, microbiological and molecular approaches we designed a multi-pronged approach to obtain clear evidence for Fe-AOM in the sediments of HMA.

**TABLE 2:** Sampling information for all gravity cores retrieved from the Helgoland Mud Area.

**Sampling objective**	**Sampling date**	**Station name**	**Coordinates**	
			**Latitude**	**Longitude**
Total Fe and Mn quantification and sequential extraction	July 2013	HE406-004-2	54° 6.03′ N	07° 59.01′ E
Total Fe quantification and sequential extraction	July 2013	HE406-008-2	54° 5.01′ N	07° 58.04′ E
Total Fe quantification and sequential extraction, pore-water profiles, *mcrA* gene sequencing and qPCR	May 2015	HE443-010-3	54° 05.19′ N	07° 58.21′ E
Long-term experiments with ^13^CH_4_ (including molecular analyses), CARD-FISH counts of active bacteria and archaea	May 2015	HE443-077-1	54° 05.23′ N	07° 58.04′ E
Pore-water profiles	April 2016	HE461-004-4	54° 05.20′ N	07° 57.99′ E
Determination AOM rates experiment	April 2016	HE461-064-1	54° 05.20′ N	07° 57.99′ E
Manganese AOM long-term experiments	April 2017	HE483-002-02	54° 05.23′ N	07° 58.04′ E

### Sampling From the Helgoland Mud Area

Sediment samples were obtained from gravity cores collected during RV HEINCKE cruises HE406 (July, 2013), HE443 (May, 2015), HE461 (April, 2016) and HE483 (April, 2017) (Table 2). As data from previous campaigns show (Oni et al., 2015; Figures 2B,C), the geochemical zonation of the sediments at the study sites are consistent over the years. Pore-water sampling of gravity cores HE443-010-3 and HE461-004-1 for dedicated geochemical analysis was done on board using rhizon samplers (Seeberg-Elverfeldt et al., 2005; Dickens et al., 2007). Sediment samples for solid-phase geochemical analysis (Table 2) were collected as described in reference (Oni et al., 2015).

Geochemical and molecular assessments were done on samples that were directly taken on board after the gears were retrieved. The gravity cores HE443-077-1 and HE483-002-2 were stored on board at 4°C and sectioned immediately (at 25 cm intervals) after the expedition and stored in the dark at 4°C in 2.6 L anoxic jars. Within 3 months after collection, sediments from HE443-077-1 were used for ^13^CH_4_ Fe-AOM incubation experiments and sediments from HE483-002-2 were used for ^13^CH_4_ Mn-AOM incubation experiments. Potential for AOM was investigated with fresh sediments from HE461-064-1 gravity core sectioned and used for ^14^CH_4_ experiments a week after core retrieval.

### Geochemical Analyses

Sulfate, hydrogen sulfide, and CH_4_ measurements were done from pore-water and sediment slurry (for CH_4_) samples as described in reference (Oni et al., 2015). Dissolved iron and manganese concentrations in pore-water were determined by inductively coupled plasma-optical emission spectrometry (Iris Intrepid II ICP-OES).

For the determination of total iron and manganese contents in the solid-phase, about 50 mg of freeze-dried and ground sediment were fully digested in a concentrated acid mixture of 3 mL HCl, 2 mL HNO_3_, and 0.5 mL HF using a CEM Mars Xpress microwave system at the Alfred Wegener Institute, Bremerhaven. Sequential extractions were performed under anoxic conditions except for the oxalate step (Henkel et al., 2016) using ∼50 mg of dry sediment and 5 mL of (a) MgCl_2_ for adsorbed Fe, (b) Na-acetate for Fe-carbonates and surface-reduced Fe(II), (c) hydroxylamine-HCl for easily reducible iron oxides (ferrihydrite, lepidocrocite), (d) Na-dithionite/citrate for reducible iron oxides (mostly goethite and hematite and some magnetite) and (e) ammonium oxalate/oxalic acid for extractable magnetite. Sequential extractions of manganese oxides were done similarly without further determination of the specific manganese mineral phases being extracted. However, the extraction gave an indication regarding the quantity of reactive manganese oxides present in the sediment. Iron and manganese analysis for bulk contents and sequential extraction solutions were performed by ICP-OES.

### Long-Term Incubations With ^13^CH_4_ Tracer

For the long-term Fe-AOM experiments, sediments from the sulfate zone (0–25 cm) and methanic zone (347–372 cm) (see Table 2) were used to set up slurry incubations. For the long-term Mn-AOM incubations, sediments from the methanic zone (195–220 cm) were used. Individual anoxic slurries were prepared by mixing 60 mL of sediments with sulfate-depleted ASW (1:3 w/v) in 120-mL serum vials. Headspace of slurries was filled with either CH_4_ (99.999%, core treatments) or N_2_ (99.999%, negative controls). Slurries were incubated at 4°C for 14 days to equilibrate the system and ensure the microcosms are completely reduced. Afterward, 15% (∼9 mL) of the headspace of CH_4_ carrying slurries was removed using an air tight syringe and replaced with 9 mL ^13^CH_4_. ^13^CH_4_ was added to the headspace to track CO_2_ formation in form of DIC within incubations from the different sediment layers and with different amendments as a proxy for AOM. From the sulfate zone sediments, treatment sets (*n* = 3) were prepared with the following modifications: (I) ^13^CH_4_ and 5 mM sodium sulfate; (II) ^13^CH_4_, 30 mM sodium molybdate and 30 mM lepidocrocite; (III) ^13^CH_4_ and 30 mM lepidocrocite; (IV) ^13^CH_4_ (V) N_2_ headspace, un-amended slurry; (VI) N_2_ headspace and 5 mM sodium sulfate; (VII) N_2_ headspace and 30 mM lepidocrocite. To slurry sets (*n* = 3) from the methanic zone, the following treatment modifications were made: (I) ^13^CH_4_ and 30 mM lepidocrocite; (II) ^13^CH_4_, 5 mM sodium molybdate and 30 mM lepidocrocite; (III) ^13^CH_4_ and 30 mM hematite; (IV) ^13^CH_4_, 5 mM sodium molybdate and 30 mM hematite; (V) ^13^CH_4_ and 30 mM magnetite; (VI) ^13^CH_4_, 5 mM sodium molybdate and 30 mM magnetite; (VII) ^13^CH_4_ and 5 mM sodium molybdate; (VIII) ^13^CH_4_ and 30 mM sodium sulfate; (IX) ^13^CH_4_; (X) N_2_ headspace, un-amended slurry; (XI) N_2_ headspace and 30 mM lepidocrocite; (XII) N_2_ headspace and 30 mM hematite; (XIII) N_2_ headspace and 30 mM magnetite; (XIV) N_2_ headspace and 30 mM sodium sulfate; (XV) ^13^CH_4_ and 30 mM birnessite; (XVI) ^13^CH_4_, 5 mM sodium molybdate and 30 mM birnessite; (XVII) N_2_ headspace and 30 mM birnessite. All iron oxides were obtained from Lanxess AG (Cologne, Germany) and their properties can be found in Supplementary Table S7. Birnessite was synthesized according to reference (Mc Kenzie, 1971). To be able to carry out lipid stable isotope probing (SIP) subsequently, we prepared several replicates for sacrificial sampling from two treatment types described above; (I) from the sulfate zone, where we expected to stimulate S-AOM (^13^CH_4_ + sulfate) and (II) from the methanic zone, where we expected to stimulate Fe-AOM (^13^CH_4_ + molybdate + lepidocrocite). All treatments for Fe-AOM were incubated at 30°C and all treatments for Mn-AOM were incubated at 10°C, sampled initially after 12–18 h (taken as time-point 0) for dissolved iron, manganese and DIC measurements and subsequently over the course of 250 days. Replicate samples for lipid SIP were also sacrificially sampled at each time-point (including time-point 0) by directly opening each serum bottle and transferring the contents into a sterile 50-mL falcon tube, which was stored immediately at −20°C until lipid extraction. Fe^2+^ and Mn^2+^ formation in aqueous phase was monitored spectrophotometrically, according to references (McArthur and Osborn, 1989; Viollier et al., 2000). For analysis of DIC isotopic composition, 2 mL of sediments from each microcosm, using syringes pre-flushed with N_2_, were transferred into 2.5-mL micro-centrifuge tubes pre-flushed with N_2_. The tubes were centrifuged at 15,300 *g* for 3 min followed by careful transfer of the supernatants into 4-mL glass vials. Vials were stored at −20°C until measurements. DIC analysis was done using a Delta Ray Isotope Ratio Infrared Spectrometer (IRIS) with URI Connect and autosampler (Thermo Fisher Scientific, Germany). As preparation of the DIC analysis, 100 μL of 45% H_3_PO_4_ was added to gas tight 12-mL exetainer vials with septum caps and flushed for 3 min with CO_2_ free air using the Delta Ray system. Afterward, 1 mL of stored liquid sample was transferred into each exetainer vial using a gas tight syringe and left for equilibration at room temperature overnight. During equilibration, the DIC components in the liquid were released as CO_2_ into the headspace due to acidification. The headspace was analyzed for carbon isotope ratio of CO_2_ as δ^13^C-DIC against CO_2_ reference gas using the Delta Ray IRIS with URI connect.

Concentrations of CH_4_ in headspace samples (100 μL) of N_2_ controls were measured on a GC (Shimadzu GC-2014, Tokyo, Japan) as described elsewhere (Aromokeye et al., 2018). CH_4_ concentrations formed in headspace were calculated using the ideal gas law with incubation temperature (30°C) as variable.

### Determination of Methane Oxidation Rates Using a ^14^CH_4_ Assay

Potential for AOM in the iron oxide-rich methanic zone and the SMT of the HMA was tested via ^14^CH_4_ AOM rate measurements. In 15 mL serum vials (*n* = 4 per treatment), 7 g of fresh sediment (50–75 cm from the SMT and 200–225 cm, 300–325 cm, 400–425 cm from the methanic zone) was anoxically homogenized (N_2_:CO_2_; 80%:20%, 152 kPa) with 7 mL sulfate-depleted artificial sea water (ASW; composition [L^–1^]: 26.4 g NaCl, 11.2 g MgCl_2_⋅6H_2_O, 1.5 g CaCl_2_⋅2H_2_O and 0.7 g KCl). Sodium molybdate (10 mM; as inhibitor of dissimilatory sulfate reduction) and CH_4_ or only CH_4_ were supplemented to the treatments. Killed controls (*n* = 4) were similarly prepared using heat inactivated sediments (autoclavation) to account for abiotic reactions. Headspace in the vials was subsequently exchanged with CH_4_ (99.999%) and the incubations were allowed to equilibrate at 10°C for 2 days, in the dark. After pre-incubation, the headspace in the vials was completely filled with CH_4_ saturated sulfate-depleted ASW. 100 μL of dissolved ^14^CH_4_ (∼24 kBq; dissolved in slightly alkaline double-deionized water) was injected into each vial and the slurries were incubated in the dark at 10°C for 8 days. Afterward, the incubation was stopped by transferring the samples into 100 mL vials containing 10 mL of 25 g L^–1^ NaOH, to fix the formed radiolabeled DIC pool as solid-phase. The concentration of applied CH_4_ was determined from headspace CH_4_ using gas chromatography (GC) coupled to flame ionization detection (FID; Focus GC, Thermo Scientific; Porapak-Q column 60/80 mesh, 4 mm length, 2 mm inner diameter). The ^14^C content of applied CH_4_ (activity) was determined by stripping and combusting the headspace CH_4_ to CO_2_ at 850°C in a combustion furnace, trapping this gas in scintillation vials containing 7 mL phenethylamine (Crill and Martens, 1986). Blanks (air) were measured to estimate background activity within the system at the end of each day. Radioactivity was measured in a liquid scintillation counter (2900TR LSA, Packard) after adding 7 mL Irgasafe Plus (Perkin Elmer, Waltham, MA, United States) scintillation cocktail. Radioactivity in the DIC pool was determined by acid digestion (Joye et al., 2004) with slight modifications. Briefly, slurries were transferred to 250-mL Erlenmeyer flasks containing an antifoam agent and few drops of bromothymol blue as pH indicator. Serum vials were rinsed with 25 g L^–1^ NaOH 2–3 times to transfer the leftover slurry. A scintillation vial containing 1 mL, 0.5 M NaOH and 1 mL phenethylamine was placed in the plastic loop and flasks were sealed with rubber stoppers to which the plastic loop was attached using a metal wire. Acid digestion was carried out by adding 6 mL of 6 N HCl by passing a needle and a syringe alongside the rubber stopper. Flasks were tightly sealed using metal clamps before shaking at 90 rpm for 4 h in order to release and trap the DIC in the scintillation vials. Radioactivity was measured as mentioned above after adding 2 mL Irgasafe Plus scintillation cocktail. AOM rates were calculated using the following equation:

AOMrate(nmolgddw-1)-1

=(C14-DIC/CH14)4×[CH]4×(1/t)×(1/g)dw

Where, ^14^C-DIC is the activity of the AOM product pool, ^14^CH_4_ is the activity of the reactant pool, [CH_4_] is the concentration of headspace CH_4_ in nmol, t represents the incubation period and g_dw_ is the dry weight of the sediment samples. Dry weight estimates were obtained from heat drying slurries as previously prepared in 50-mL tubes at 80°C for 48 h. Final rates were calculated after deducting rates measured in killed controls.

### Molecular Analyses of Sediments and ^13^CH_4_ Tracer Experiments

#### Nucleic Acid Extraction

Aliquots of sediments were sampled depth-wise directly on board during pore-water sampling from HE443-010-3 gravity core (Table 2) and were immediately frozen at −20°C. Using these sediment samples, DNA was extracted from 0.5 g of sediment per depth in duplicates following the phenol-chloroform-isoamylalcohol method (Lueders et al., 2004). Similarly, nucleic acids were extracted at specific time-points (day 0, 120 or 144 and 250) in the ^13^CH_4_ experiments. Here, ∼0.5 g sediment pellets, which were stored during sampling from biological triplicates samples of each treatment, were used for the extraction (pore-water previously extracted for DIC measurement). 50 μL of diethyl pyrocarbonate (DEPC) treated water was added to elute nucleic acids from the first sample replicate. This eluent was subsequently transferred to other sample replicates in order to have the nucleic acids pooled together in one tube.

#### Next Generation Sequencing of *mcrA* and 16S rRNA Genes

Using polymerase chain reaction (PCR) method, *mcrA* genes were amplified from DNA extracts from sediment samples taken from HE443-010-3 gravity core. The primer pairs mlasF (5′-GGTGGTGTMGGDTTCACMCARTA-3′) (Steinberg and Regan, 2008) and ME2mod (5′-TCATBGCRTAGTTNGGRTAGT-3′) (Mori et al., 2012) were used for the amplification. DNA was amplified using AmpliTaq DNA polymerase kit (Thermo Fisher Scientific, Germany) containing 1X PCR buffer, 0.2 mM dNTP mix, 1.5 mM MgCl_2_, 0.2 mg mL^–1^ bovine serum albumin (BSA), 500 nM of each primer, 1U of AmpliTaq DNA polymerase and 2 μL of diluted DNA in a 50 μL reaction volume (final volume made up with DEPC treated water). Amplification was done at the following PCR conditions: 95°C: 5 min; 30 cycles at 95°C: 30 s, 50°C: 45 s, 72°C: 45 s and 72°C: 5 min. Amplicons were screened on gel electrophoresis (2% Agarose, 100V, 60 min) and purified using the QIAGEN MinElute kit (QIAGEN, Hilden, Germany) following the manufacturer’s instruction. Purified amplicons were sent to MR DNA (Molecular Research LP, Shallowater, TX, United States) for sequencing on an Illumina MiSeq (2 × 300 bp) sequencing platform.

Bacterial and archaeal 16S rRNA genes were amplified from DNA extracts from the ^13^CH_4_ incubation experiments using Illumina HiSeq 4000 (2 × 150 bp) amplicon sequencing platform. Primer pairs Bac515F (5′-GTGYCAGCMGCCGCGGTAA-3′) (Parada et al., 2016) and Bac805R (5′-GACTACHVGGGTATCTAATCC-3′) (Herlemann et al., 2011) were used for targeting bacteria, whereas Arc519F (5′-CAGCMGCCGCGGTAA-3′) (Ovreås et al., 1997) and Arc806R (5′-GGACTACVSGGGTATCTAAT-3′) (Takai and Horikoshi, 2000) were used for targeting archaea. Each primer was synthesized with an additional unique barcode sequence (8 bp long) that facilitated multiplexing of several samples in one sequence library (Hamady et al., 2008). PCR reaction mix (50 μL) contained 1 x KAPA HiFi buffer, 0.3 mM dNTP mix, 0.25 U KAPA HiFi DNA polymerase (KAPA Biosystems, Germany), 1.5 μM each of forward and reverse barcoded primer pairs, and 2 μL of 10-fold diluted DNA template from each sample (volume made up to 50 μL using DEPC treated water). PCR cycling conditions include: 95°C: 5 min; 28 cycles at 98°C: 20 s, 60°C: 20 s, 72°C: 20 s; 72°C: 1 min. PCR products were screened by gel electrophoresis as mentioned before and purified using Monarch^®^ PCR & DNA purification kit (New England Biolabs, Germany). PCR products were quantified using Quant-iT PicoGreen dsDNA assay kit (Invitrogen-Thermo Fischer Scientific, Steinheim, Germany). Based on the estimated quantities from the PicoGreen assay, an equimolar library of samples was constructed. Amplicon library was sequenced at GATC Biotech GmbH, Germany.

#### Cloning and Quantification of *mcrA* Genes of Anaerobic Methane Oxidizing Archaea (ANME)

Due to the unavailability of cultured strains of methanotrophic archaea, we cloned genes obtained from HMA sediment samples to use them as standards for quantifying gene copy numbers of ANMEs. DNA extracts from different depths of the sediments [HE376-007-5 (Oni et al., 2015); HE443-10-3: this study] were amplified using the primer pairs (I) mcrA-312f (5′ CAACBCNGCVATGCAGCAG 3′, this study)–ME2mod and (II) mlasF-ME2mod using the AmpliTaq DNA polymerase kit (same as before). The following PCR program was used for the mcrA-312f-ME2mod primer pairs: 95°C: 5 min; 30 cycles at 95°C: 30 s, 55°C: 1 min, 72°C: 1.5 min; and 72°C: 5 min. PCR products were purified using QIAGEN MinElute kit following the manufacturer’s instructions. Purified PCR products were cloned, sequenced and edited as described (Oni et al., 2015). An in-house *mcrA* gene database was created by acquiring^1^ and manually aligning long (>1000 bp) gene sequences of cultured and published methanogenic, methanotrophic and hydrocarbon degrading archaea in ARB 6.02 (Ludwig et al., 2004). Using the RAxML algorithm in ARB, a phylogenetic tree was constructed, to which shorter *mcrA* gene sequences were added using the ARB Parsimony tool. Edited FASTA sequences were imported and translated into their protein sequences in ARB. The protein sequences of clones were manually aligned and imported in the aforementioned *mcrA* gene database using the ARB Parsimony tool in order to determine their taxonomic affiliations (on DNA and protein level). Abundances of specific ANME phylotypes were determined from various sediment depths and from ^13^CH_4_ tracer experiments using quantitative PCR (qPCR). In order to estimate increase in biomass of ANME phylotypes in the incubations experiments over 250 days, abundances from their respective depths were considered as baseline proxy. qPCR assay was done following reference (Reyes et al., 2017) with few modifications. Standard templates were prepared by amplifying ANME clones using plasmid specific M13 primer pairs and the AmpliTaq DNA polymerase kit. PCR products were then purified (QIAGEN MinElute kit) and quantified using Quant-iT PicoGreen dye. Takyon ROX SYBR 2X MasterMix (Eurogentec, Seraing, Belgium) was used as a replacement kit instead of the MESA BLUE qPCR kit for the SYBR qPCR assay as recommended by the company. DNA extracted from the incubations and *in situ* sediment samples was quantified using Quant-iT PicoGreen dye and diluted to 500 pg/μL (standards and sulfate zone incubations) and 50 pg/μL (methanic zone incubations). Two μL of diluted DNA was used as template for all qPCR assays. qPCR assays were run using the following program: 95°C: 10 min; 40 cycles at 95°C: 30 s, 52°C or 62°C: 20–30 s, 72°C: 40 s. A post amplification melting curve analysis was performed in order to rule out PCR by-products by detecting change in fluorescence every 0.5°C from 60°C to 95°C. qPCR primers, assay conditions, efficiencies and clone information are provided in Table 3.

**TABLE 3:** Supporting information for phylotype specific qPCR assays.

**Target *mcrA* gene**	**Annealing temperature/time**	**Average efficiency**	***R*^2^**	**Clone used**	**Mass of one gene (Da)**	**Primer sequences (5′ - 3′)**	**References**	**Primer concen- tration**	**Product length (bp)**
ANME-1	62°C/30 s	84.45%	>0.99	E-3	878414	F: AYGACCAGYTGT GGTTCGGAACGT	Miyazaki et al., 2009	600 nM	175 bp
						R: TCCATGTTSARC TTGTCGCCCTTY			
ANME-2a	62°C/30 s	84.96%	> 0.99	F-79	862362	F: ATATGGCAGATATTG TCCAGACCTCAAGG		600 nM	218 bp
						R: ATTTATCCC AKCCGTAYTC			
ANME-3	52°C/20 s	85.67%	>0.984	AII58	440445	F: AAGGAYATYRS AACCGAATC		400 nM	180 bp
						R: TTGAAAGGTACC ATSSKGAAAGACC			
ANME-1- related	52°C/20 s	89.08%	>0.99	E-155	870491	F: GAGATCGCVRTV GACATGTTCGG	Zhou et al., 2014	400 nM	172 bp
						R: GCCCTMACAG AMCCRCCGAAGTG			

#### Analysis of *mcrA* and 16S rRNA Gene Sequences

Sequence analysis was performed on the QIIME 1.8.0 platform (Caporaso et al., 2010) based on the analysis pipeline as recommended (Pylro et al., 2014) with modifications. To analyze *mcrA* gene sequences, barcodes were extracted and sequences were reoriented starting with the forward primer sequence. Reoriented reads were joined using a minimum overlap of 50 bases. Joined reads were demultiplexed with a filter quality of Q0 (Caporaso et al., 2011). Demultiplexed sequences were quality filtered using USEARCH 10 (expected error value of 0.5) (Edgar, 2010). At this step, all sequences were truncated to a length of 352 bp. USEARCH 10 was further used to dereplicate sequences, sort them by their abundances and subject them to remove singletons. OTU clustering and chimera removal was done using the UPARSE-OTU algorithm (Edgar, 2013) to create an OTU database. Chimeric sequences were checked and discarded by the UPARSE-OTU algorithm during this step. The truncated, non-dereplicated reads were mapped back to the OTU database to create an OTU table. OTUs were classified for their taxonomy using uclust and an in-house *mcrA* gene database as reference (see *mcrA* genes cloning section). The taxonomic assignment was done on the family level at a sequence identity of 0.7 (Yang et al., 2014). The OTU table and taxonomy assignment files were merged together using a set of “biom” commands (McDonald et al., 2011) to obtain a tab-delimited text file useful for downstream analysis. A few modifications of the above pipeline were done to analyze 16S rRNA gene sequences. Forward reads were used to analyze the community composition. After extraction of barcodes, forward reads were de-multiplexed, quality filtered and their lengths were truncated to 143 bp. Taxonomic assignment was done on clustered OTUs against the 16S rRNA gene SILVA database (Release 128 for QIIME) (Quast et al., 2012).

#### Cloning and Amplification of *pmoA* Gene

To maximize amplification of *pmoA* genes, a two-step PCR was conducted with AmpliTaq DNA polymerase kit with slight modifications compared to the *mcrA* gene amplification (1.25U of AmpliTaq polymerase and 3 mM MgCl_2_ were used instead). Primer pairs of A189F (5′-GGNGACTGGGACTTCTGG-3′) (Holmes et al., 1995) and 682R (5′-GAASGCNGAGAAGAASGC-3′) (Holmes et al., 1995) were used for the first PCR at the following conditions: 94°C: 4 min; 35 cycles at 94°C: 30 s, 50°C to 60.5°C (0.3°C per cycle) and 72°C for 1 min; 72°C: 7 min. PCR products were purified using the QIAGEN MinElute kit. Purified products were further amplified using primer pairs A189F and mb661R (5′-CCGGMGCAACGTCYTTACC-3′) (Smith et al., 1997) at the following conditions: 94°C: 4 min; 35 cycles at 94°C: 30 s, 55°C: 1 min and 72°C: 1 min; 72°C: 1 min. Cloning was done (see *mcrA* gene cloning) to identify the unspecific PCR products of ∼300–350 bp from the *pmoA* gene PCR amplification (Supplementary Figure S10). PCR products from ^13^CH_4_ + molybdate + magnetite (P) and ^13^CH_4_ + molybdate + lepidocrocite (L) incubations were purified, cloned and sequenced as per mentioned in *mcrA* gene cloning section. Using BioEdit (Hall, 1999) (version 7.0.9.0), vector sequences were trimmed and the sequences were reoriented (if necessary) by locating the A189F primer sequence. A sorted six-frame translation of the nucleotide sequences was performed in BioEdit and the longest amino acid sequences (110 amino acids; without a stop codon) in the positive frame were selected and stored in the FASTA file format. Amino acid sequence FASTA files were uploaded to the Protein BLAST suite (blastp suite^2^) and BLAST hits were tabulated in Supplementary Table S5.

#### Bacterial and Archaeal Cell Counts Using Catalyzed Reporter Deposition-Fluorescence *In situ* Hybridization (CARD-FISH)

CARD-FISH was performed as previously described (Schmidt and Eickhorst, 2014) to quantify potentially active bacterial and archaeal cells at different sediment depths (HE443-077-1). Approximately 0.5 g of sediment samples were weighed in 2-mL vials and were fixed in 4% formaldehyde and 1X phosphate-buffered saline. The 2-mL vials were incubated at 4°C for 2.5 h under constant shaking (180 rpm on an overhead shaker). The suspension was centrifuged and the sediment was washed with 1.5 mL of 1X PBS twice. After decanting the supernatant, fixed samples were re-suspended using 1.5 mL 1X PBS:ethanol (v:v) solution. 100 μL of fixed samples were transferred to 900 μL of 1X PBS:ethanol (v:v) solution and sonicated in pulses at 10% power for 30 s (two times paused by 30 s) in a cryo-box. Two hundred fifty μL of sonicated samples were mixed with 10 mL of Milli-Q water (H_2_O_MQ_) and vacuum filtered through a polycarbonate filter (0.22 μm) in order to capture fixed cells on the filter. After air drying, the filter was dipped in molten, 0.2% low melting point Agarose and allowed to dry at 46°C. Permeabilization of the cell walls was done using 100 μL solutions of lysozyme (60 min, 37°C) and achromopeptidase (30 min, 37°C). The filters were washed with H_2_O_MQ_ after each treatment. Inactivation of endogenous peroxides, hybridization with horseradish peroxidase labeled probes, washing of unbound probes and tyramide signal amplification (with 500 μL amplification buffer) was done as mentioned in reference (Schmidt and Eickhorst, 2014). Bacterial cells were targeted with a mixture of three probes namely, EUB338 (5′ GCTGCCTCCCGTAGGAGT 3′) (Amann et al., 1990), EUB338II (5′ GCAGCCACCCGTAGGTGT 3′) (Amann et al., 1990) and EUB338III (5′ GCTGCCACCCGTAGGTGT 3′) (Daims et al., 1999). Archaeal cells were targeted with the ARC915 probe (5′ GTGCTCCCCCGCCAATTCCT 3′) (Amann et al., 1990). The filters were mounted on a clean glass slide containing a drop of VectaShield H-1200 containing DAPI in order to counter-stain cellular DNA. Cells were observed and counted following reference (Schmidt and Eickhorst, 2014).

### Lipid SIP

^13^C incorporation into bacterial and archaeal membrane lipids was monitored during the ^13^CH_4_ incubations. Sacrificed replicates from each time-point were used to investigate changes in stable carbon isotopic composition of the lipids. While bacterial lipids were continuously monitored over the whole incubation period, archaeal lipids were only determined at the start and the end.

Total lipids were extracted from freeze-dried slurries (10–12 g) following a modified Bligh and Dyer method (Sturt et al., 2004). Afterward, polar lipid derived fatty acids (PLFAs) of bacteria were released from aliquots (30%) of the total lipid extract (TLE) and converted into fatty acid methyl esters (FAMEs) (Elvert et al., 2003). Intact and core archaeal lipids from a second TLE aliquot (30%) were separated by preparative HPLC and ether lipids in each fraction were converted to hydrocarbons (Lin et al., 2010). Separation was achieved by a LiChrosphere Diol-100 column (250 × 10 mm, 5 μm particle size, Alltech) connected to an Agilent 1200 series HPLC that was equipped with an Agilent 1200 series fraction collector, at 30°C, with a flow rate of 3 mL min^–1^. The eluent gradient was 0–24% B in 15 min, to 100% B in 5 min and hold for 10 min where eluent A was composed of *n*-hexane and isopropanol (IPA) (90/10; v/v) and eluent B of 100% IPA. FAMEs and ether-cleaved hydrocarbons were subsequently analyzed by a Thermoquest Trace GC mass spectrometry (GC-MS) system and GC-isotope ratio-MS (GC-IRMS) using a Trace GC ultra-coupled via GC Isolink and a Conflo IV interface to a Thermo Scientific Delta V plus following published protocols (Kellermann et al., 2012).

Uptake of ^13^CH_4_ into PLFAs was calculated as the product of excess ^13^C and the amount of PLFA carbon based on quantification via GC-FID measurements (Kellermann et al., 2012). Excess ^13^C is the difference between the fractional abundance (F) of ^13^C in PLFAs after 250 days relative to the T_0_ sample where F = ^13^C/(^13^C + ^12^C) = R/(R + 1), with R being derived from the measured δ^13^C values as R = (δ^13^C/1000 + 1) × R_VPDB_.

### Statistical Analysis and Figures Production

Correlation analysis and figures were made within the R environment (R Core Team,, 2019). Pearson correlation coefficients (*r*) were calculated along with confidence intervals (95%). P values were adjusted for multiple testing (False Discovery Rate method).

## Data Availability Statement

Raw sequence data used in this study can be accessed from GenBank Short Reads Archive with accession number SRP156177. Clone sequences used in this study for qPCR were deposited to GenBank and have been assigned the accession numbers MH917693–MH917696. Geochemical dataset was submitted to the PANGAEA data publisher for Earth & Environmental Sciences database under the following doi https://doi.pangaea.de/10.1594/PANGAEA.893768.

## Author Contributions

DA, AK, OO, ME, GW, SK, and MF designed the study. SH, SK, DA and AK performed geochemical sampling and analysis. GW, AK, and DA performed ^14^CH_4_ experiments. AK, DA, and TE performed molecular biology assessments on sediment samples. DA, OO, HT, and AK performed the ^13^CH_4_ incubation experiments. AK, DA, XY, and LW performed molecular biology assessments on the ^13^CH_4_ incubations. DA, QZ, SC, and ME performed lipid stable isotope probing on the ^13^CH_4_ incubations. AS, XY, and HT performed manganese AOM incubation experiments. TR-H performed the figures production and statistical analysis with support from AK and DA. GW, ME, K-UH, SK, and MF obtained funding for this research. DA and AK contributed equally and together with MF wrote the article with contribution from all co-authors.

## Conflict of Interest

The authors declare that the research was conducted in the absence of any commercial or financial relationships that could be construed as a potential conflict of interest.

## Footnotes

1 https://www.ncbi.nlm.nih.gov/nucleotide/

2 https://blast.ncbi.nlm.nih.gov/Blast.cgi
