# Supplementary material for: Rates and Microbial Players of Iron-Driven Anaerobic Oxidation of Methane in Methanic Marine Sediments
Source: Front Microbiol. 2020 Jan 17;10:3041. doi: 10.3389/fmicb.2019.03041 (PMC6979488; doi:10.3389/fmicb.2019.03041)
Supplement: Supplementary file 1 [file Data_Sheet_1.docx]

Supplementary Material

**Supplementary Results and Discussion**


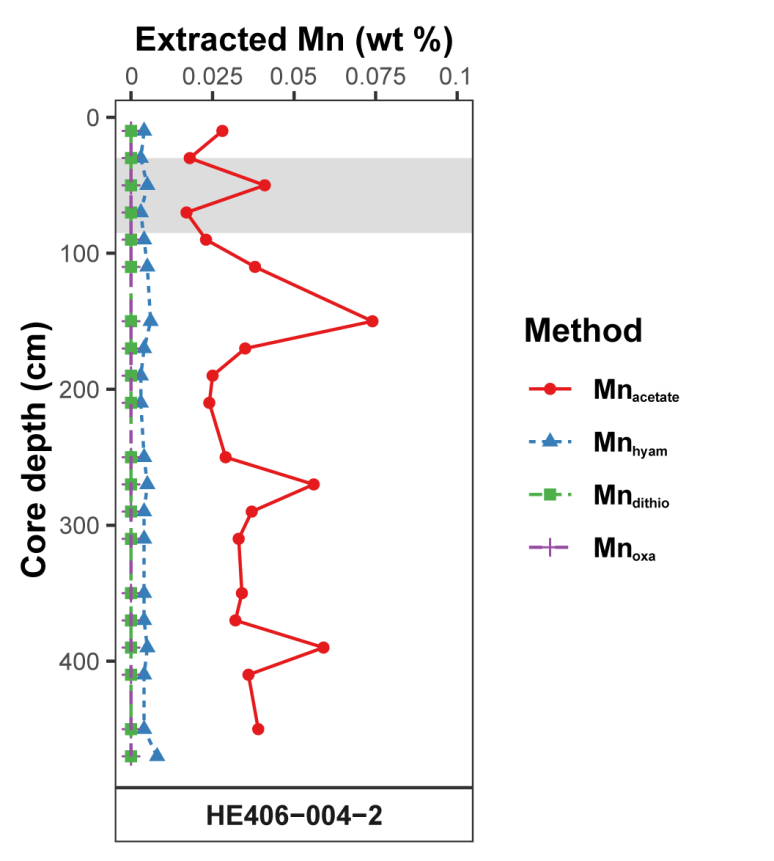


**Figure S1.** Operationally defined manganese oxide phases in Helgoland Mud Area obtained from HE406-004-2 gravity core. (Mn_acetate_: sodium acetate extractable, Mn_hyam_: hydroxylamine-HCl extractable, Mn_dithio_: dithionite extractable and Mn_oxa_: oxalate extractable manganese oxide phases). Grey area represents the SMT.

| 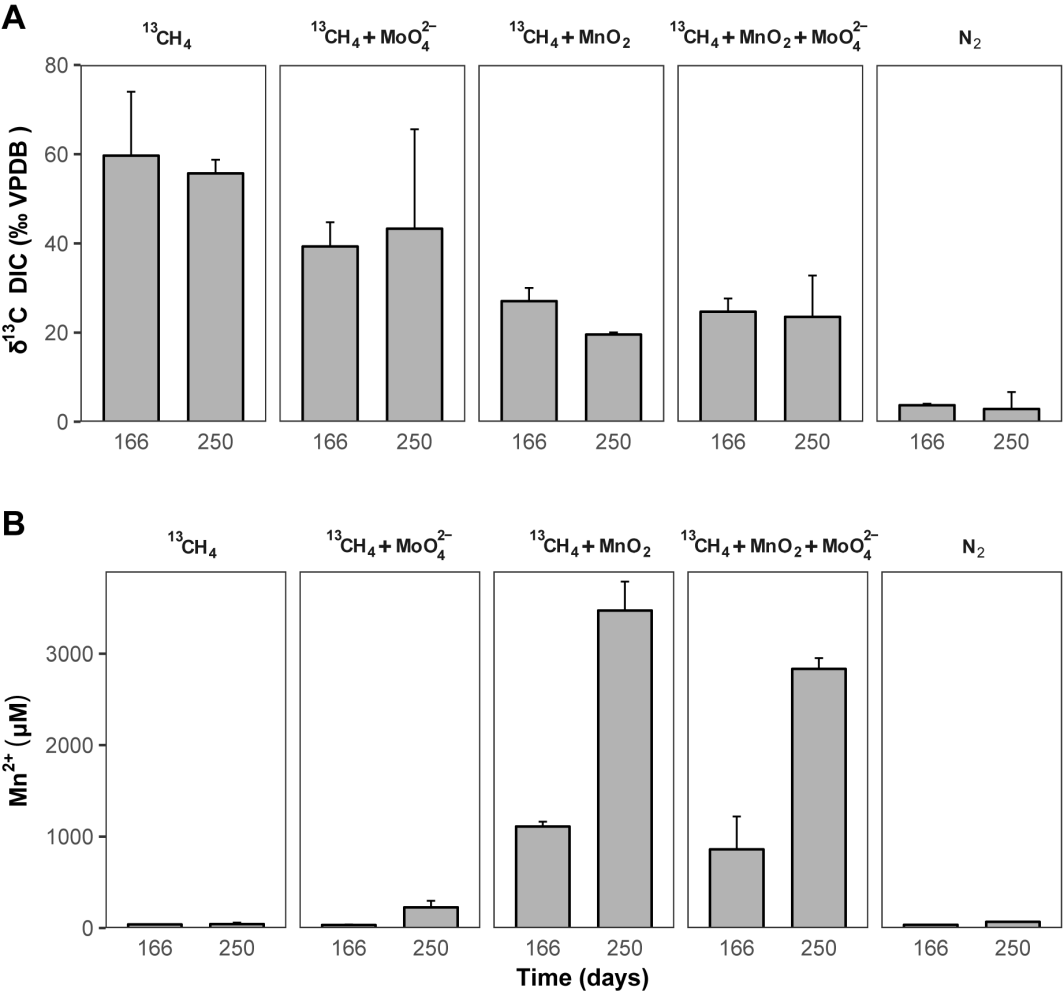  **Figure S2.** Manganese dependent AOM in slurry incubations from methanic zone sediments (195-220cm) of Helgoland Mud Area. **(A)** Changes in δ^13^C DIC values after 166 and 250 days. **(B)** Changes in Mn^2+^ concentration after 166 and 250 days. n = 3, error bars represent 1 s.d. of biological replicates. AOM rates were less in the MnO_2_ amended incubations compared to controls without MnO_2_ showing the suppressive effect of manganese oxide on AOM in the methanic zone unlike iron oxides (Figure 4A). |
| --- |

**
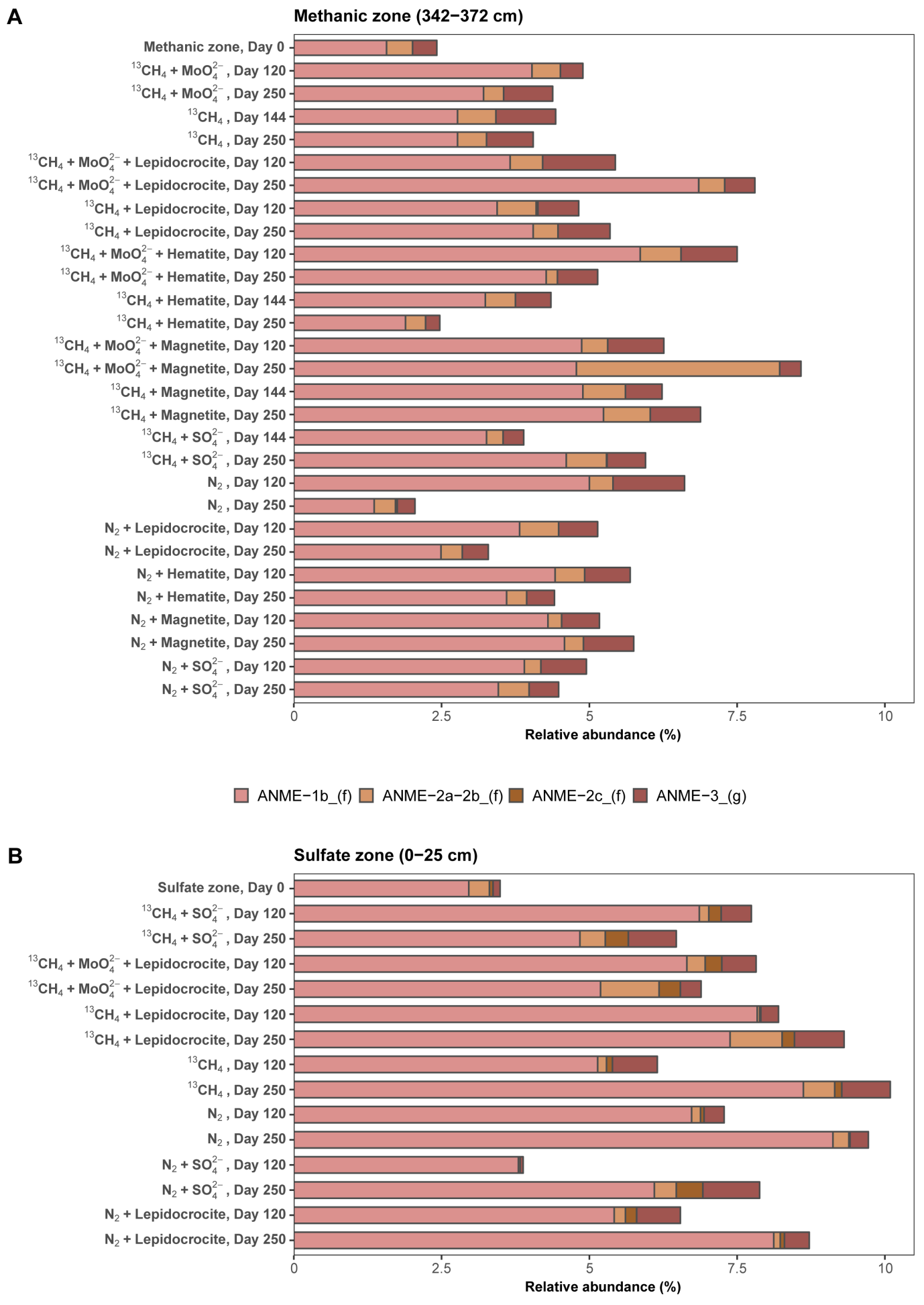
**

**Figure S3.** 16S rRNA genes belonging to known ANME as a fraction of total archaeal 16S rRNA genes in incubations of both geochemical zones.


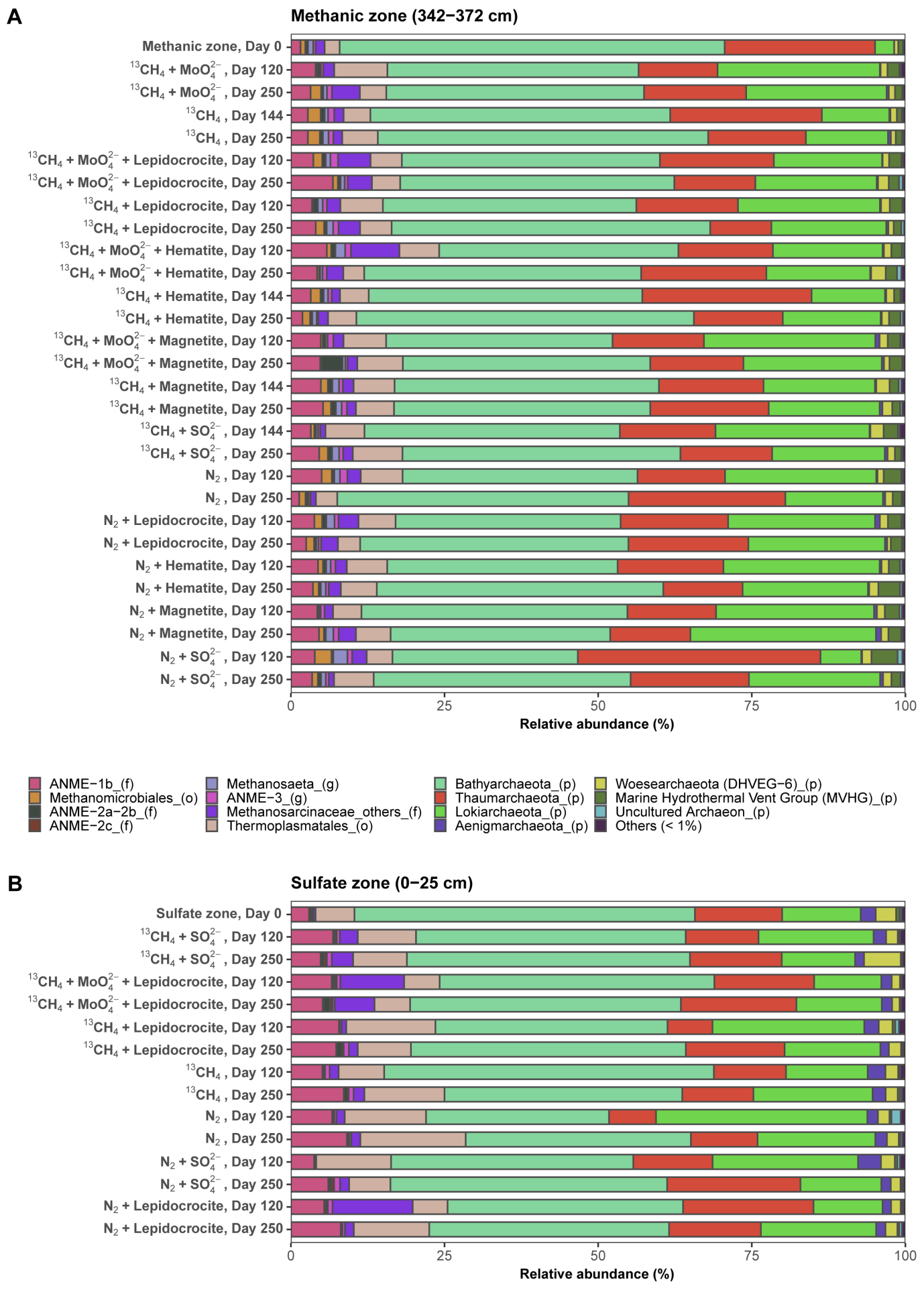


**Figure S4.** Total sum scaling of archaeal 16S rRNA genes across all incubations from both geochemical zones.

| 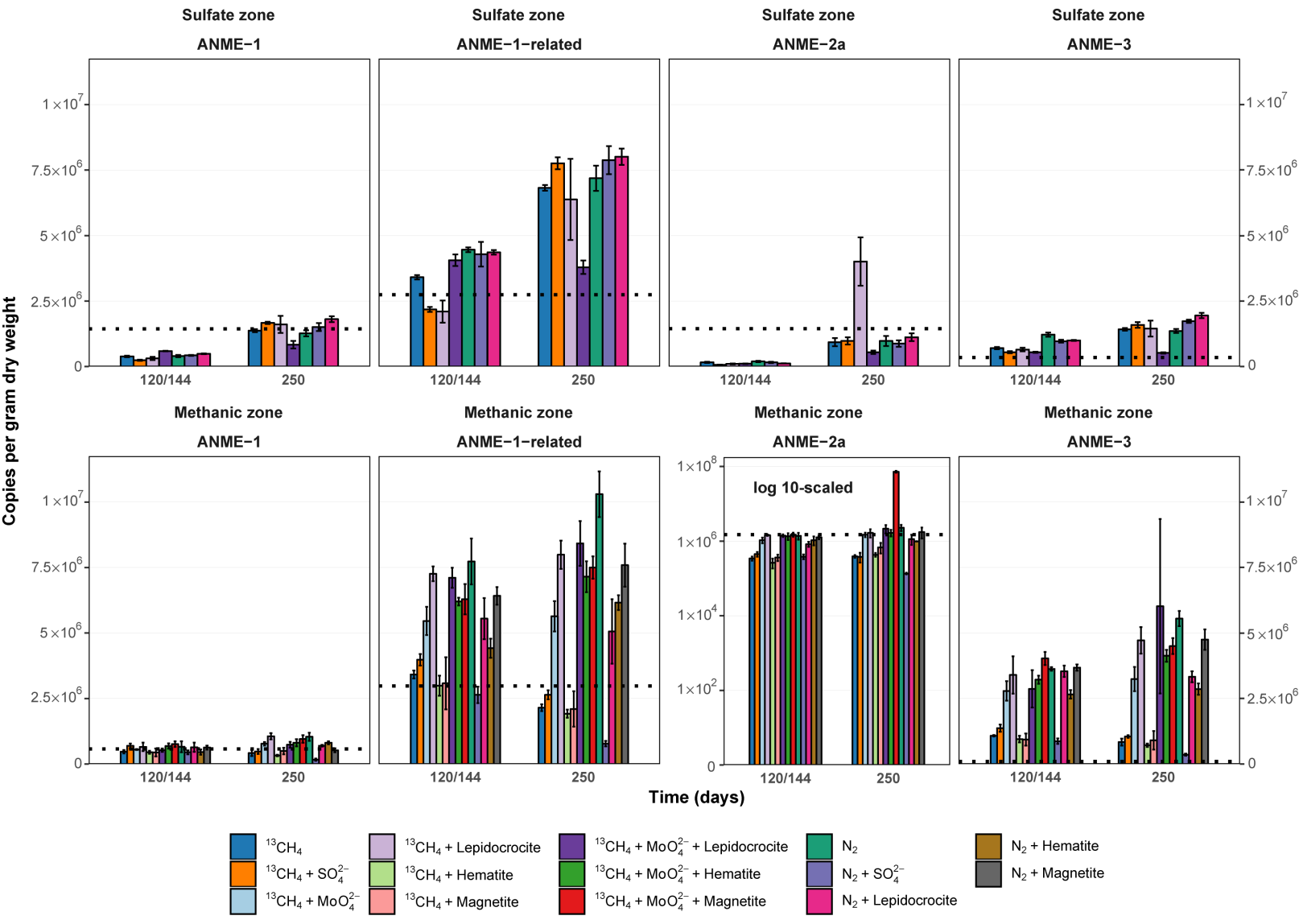  **Figure S5.** *mcrA* gene qPCR results of ANMEs from all incubations after either 120 or 144 days as intermediate time-point and after 250 days of incubation. Top panel represents incubations from the sulfate zone and bottom panel represents incubations from the methanic zone. Gene copy numbers were compared with copy numbers from the respective depths (grey baselines) used for setting up the experiment in both geochemical zones. |
| --- |

**CH_4_ in the headspace of N_2_ controls**

The microbial community composition dataset shown in Figures S3–S5 showed the presence and stimulation of ANMEs in N_2_ filled microcosm controls as well. Therefore, CH_4_ in the headspace of controls was measured to check for CH_4_ production in the incubations (Figure S6). Since CH_4_ production was observed in the control incubations the amount of CH_4_ found in the headspace potentially fueled the survival of ANMEs in the N_2_ controls.

| 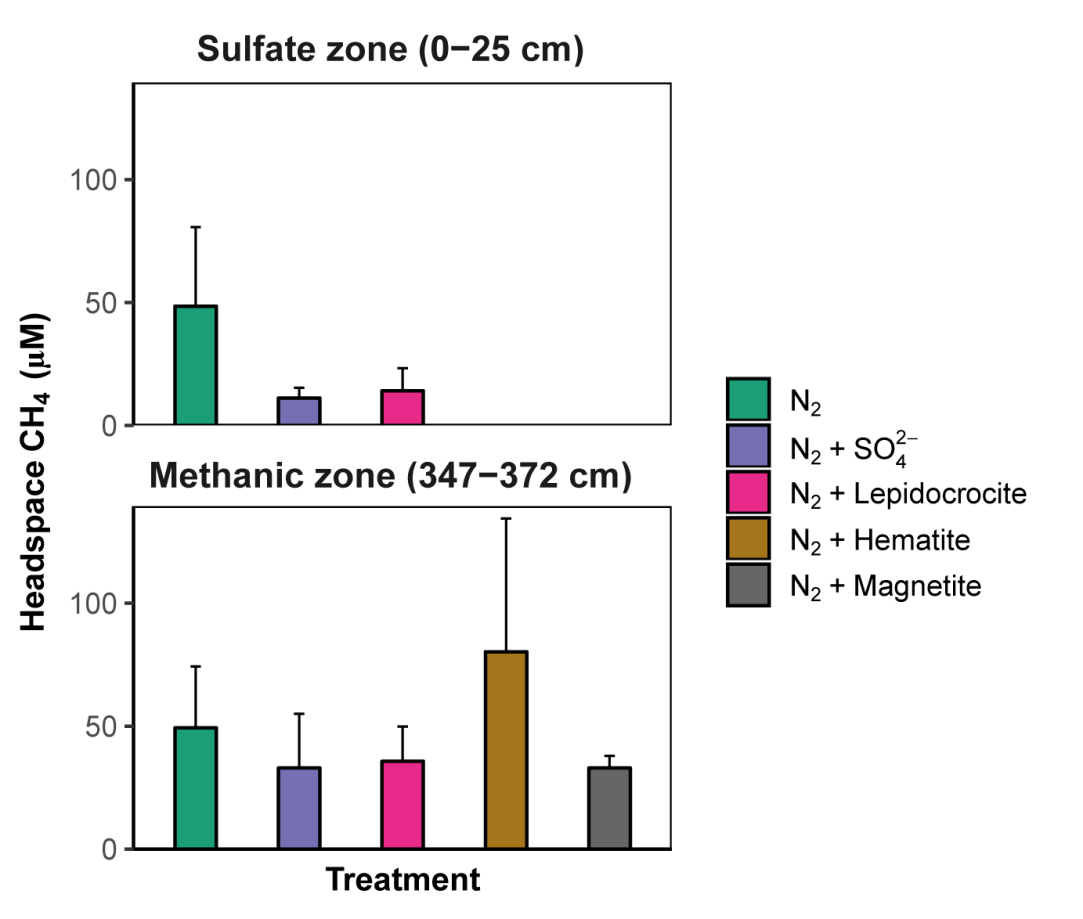  **Figure S6.** CH_4_ in the headspace of N_2_ controls after ~ 500 days. |
| --- |

Although additional carbon source was not added to the sediments, inherent organic matter in the sediments fueled CH_4_ production.


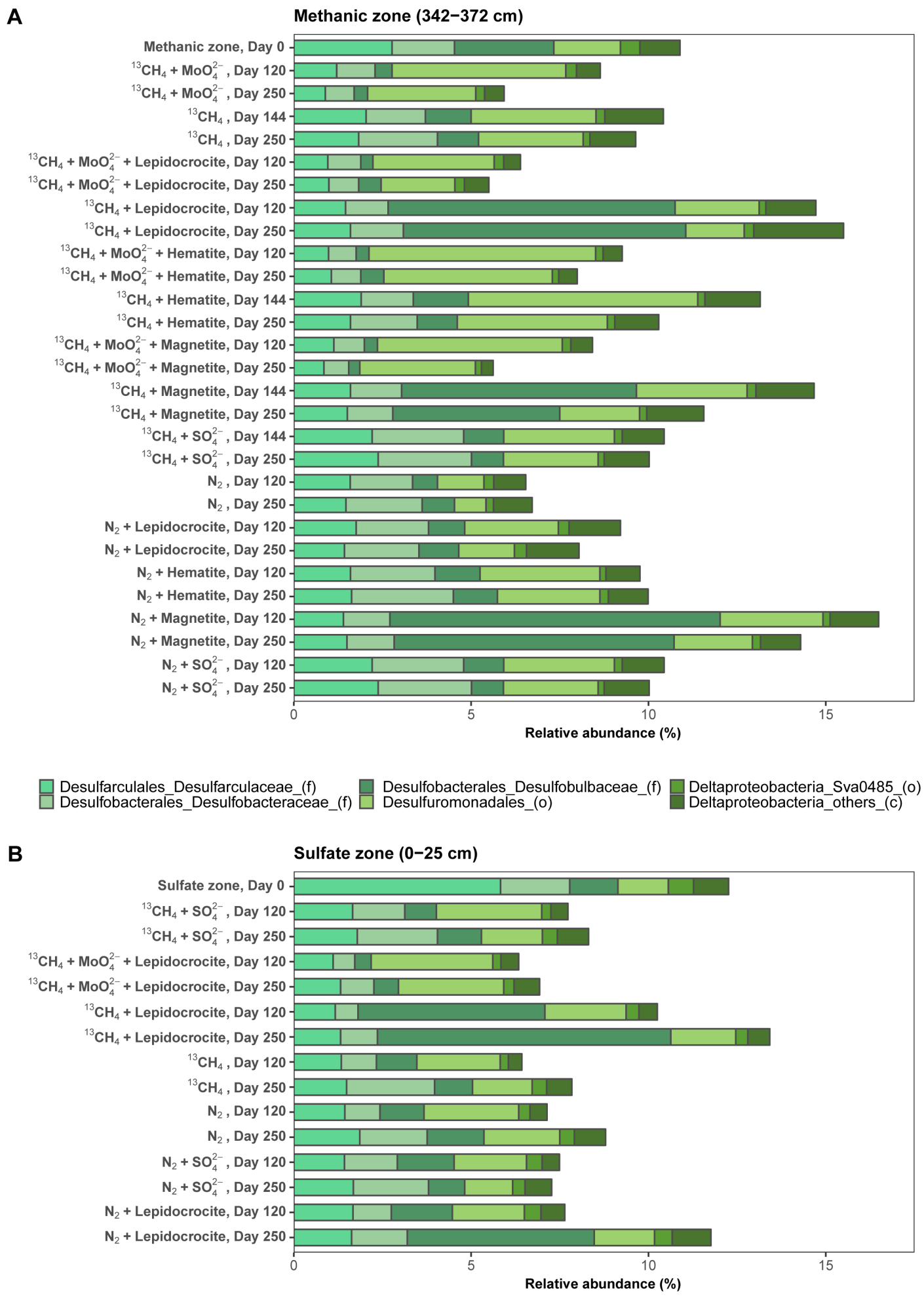


**Figure S7.** 16S rRNA sequences belonging to Deltaproteobacteria across all incubations. Sequences belonging to dissimilatory iron reducing family Desulfuromonadales were more abundant, relative to known sulfate reducers, in incubations with molybdate amendment. This indicated that Fe-reducers were predominant in these incubations due to Fe-AOM.

**Dissolved Fe^2+^ measurement across all incubations**

As shown in Figure 2B, 2C, FeCarb phases, which are representative of Fe(II) phases, are abundant in the sediment. Given that the incubation experiments were carried out using natural sediment samples replete with solid phase Fe(II) fractions, measurement of HCl extractable Fe(II) would potentially leach out Fe(II) fractions already present in the sediment. Consequently, this overprints on the freshly produced Fe(II) from ongoing microbial iron reduction. Therefore, dissolved Fe^2+^ in aqueous phase was measured in real time and directly used as proxy for rates of microbial iron oxide reduction (Figure S8). While lepidocrocite amended incubations showed the highest concentrations of dissolved Fe^2+^, magnetite amended incubations in the methanic zone showed the lowest concentrations of dissolved Fe^2+^ in the headspace.


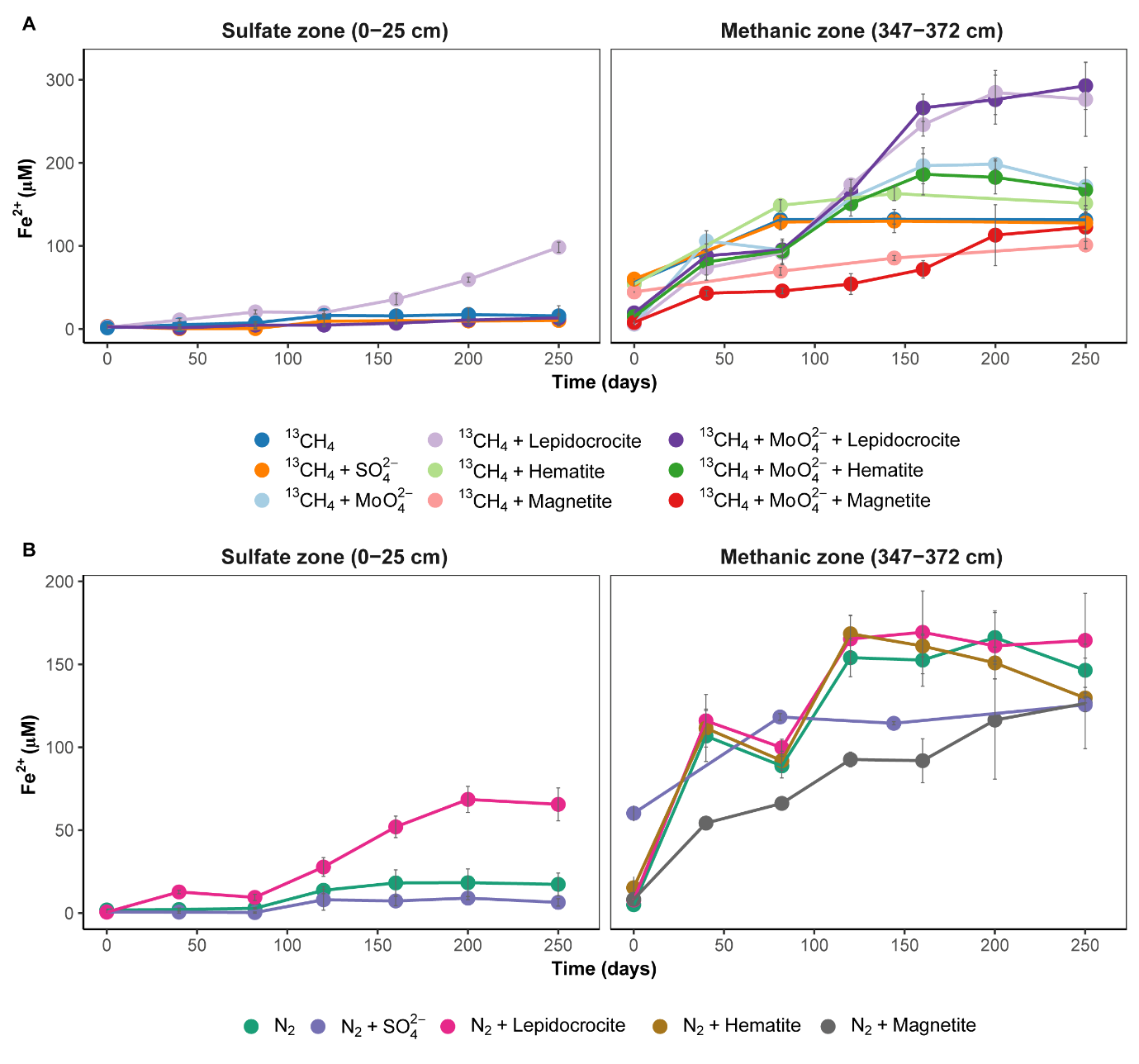


**Figure S8.** Dissolved Fe^2+^ measurements over time in incubations with **(A)** ^13^CH_4_ in the headspace and **(B)** control incubations with N_2_ in the headspace.

Although the Fe^2+^ concentrations formed in N_2_ amended control incubations (Figure S8B) are slightly lower than in parallel incubations with ^13^CH_4_ in the headspace (Figure S8A), the results show that there are potentially other pathways generating Fe^2+^ in the sediment, mostly due to organic matter in the sediments. The fact that iron oxide reduction in these incubations cannot be totally attributed to Fe-AOM alone shows that these Fe^2+^ data cannot be used for an accurate stoichiometric assessment of Fe-AOM. Future enrichments of sediment free communities from these sediments will allow for such assessments.

| 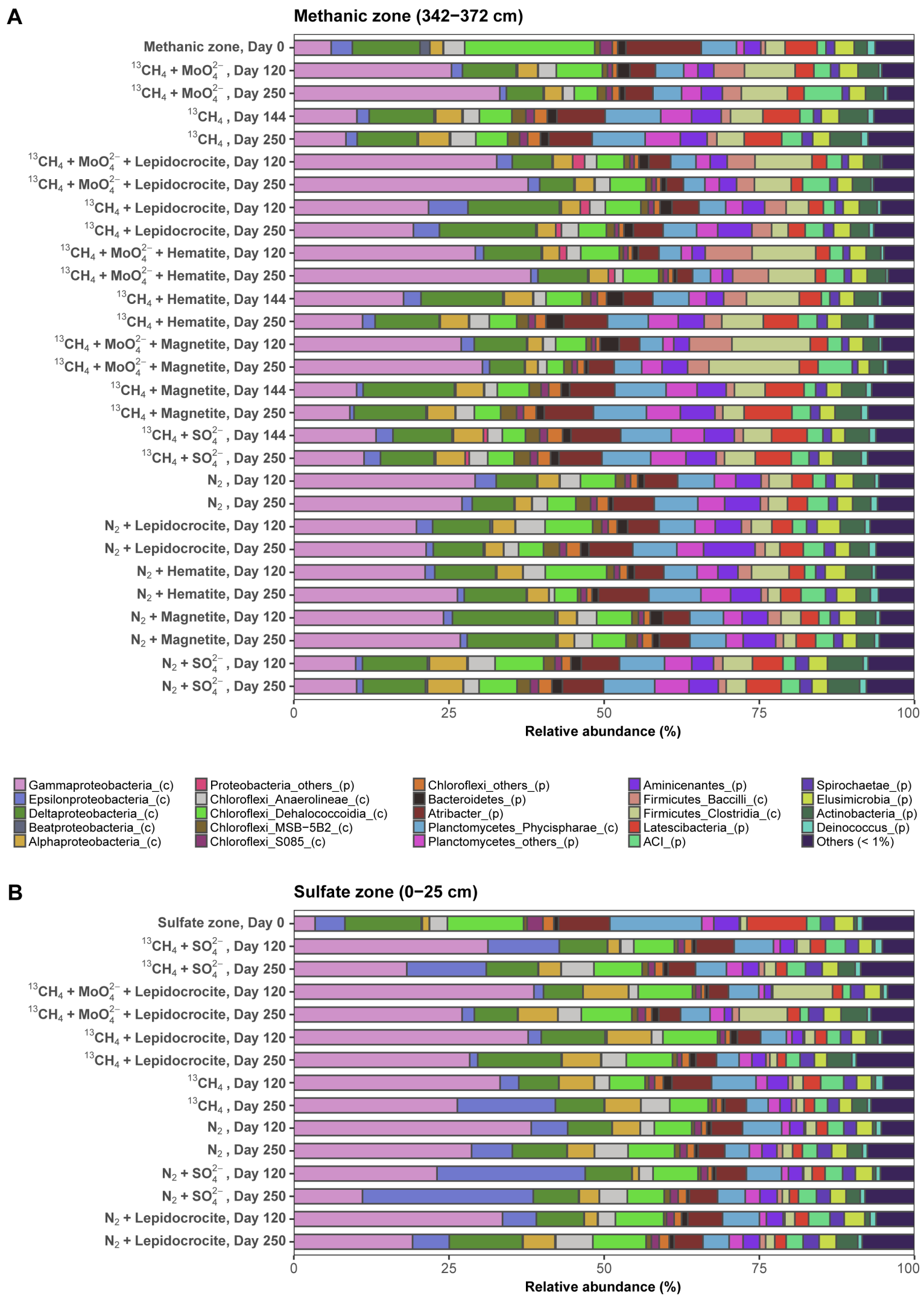  **Figure S9.** Total sum scaling of bacteria 16S rRNA genes across all incubations from both geochemical zones. |
| --- |

| ***pmoA* gene analysis and bacteria PLFA results reveal the absence of aerobic methanotrophs in our incubations**  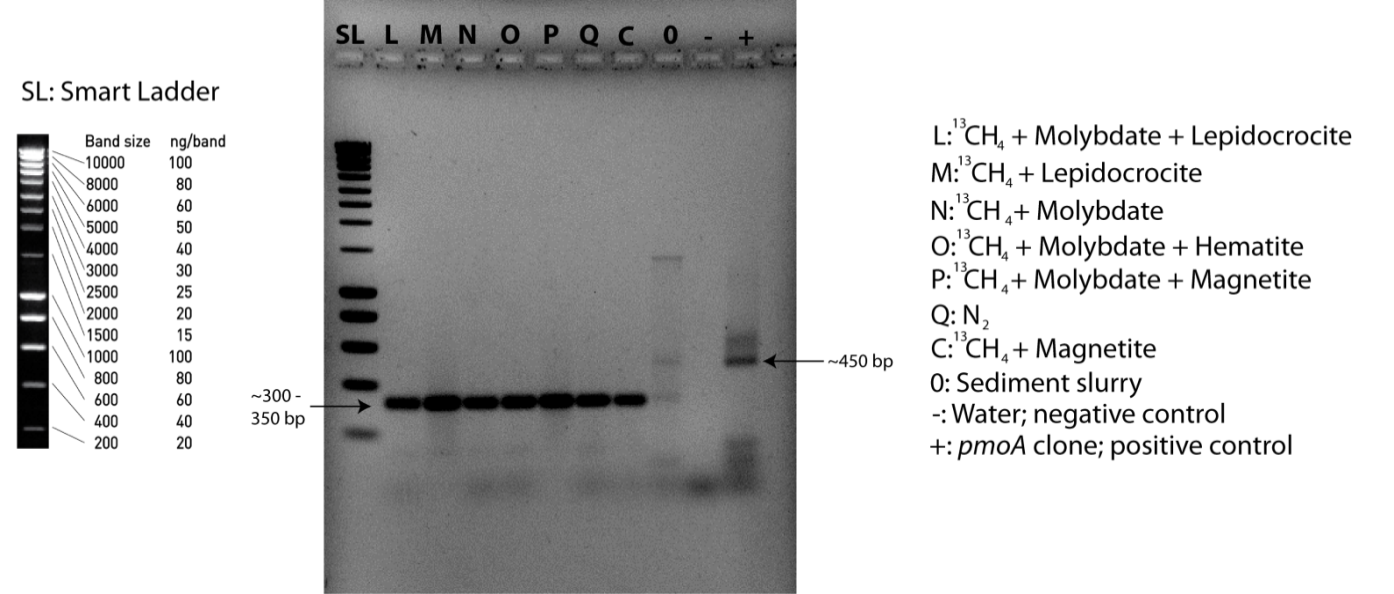Given the recent evidence that methanotrophic bacteria are involved in Fe-AOM, we carried out two-step pmoA PCR (see methods section) to check for the presence of pmoA functional gene in our methanic zone treatments. Expected length of PCR product (472 bp) was only found in the initial sediment slurry used for the incubation experiment and not in the treatments after 250 days (Figure S10); however, there was an unspecific amplification at 330 bp whose identity was checked by cloning. The cloned sequences were not closely related to any known methanotrophic bacteria (Table S5) or pmoA gene.   \| **Figure S10.** Unspecific amplification of the pmoA gene from incubations (L–Q and C) from the methanic zone. \| \| --- \| |
| --- | --- |

**DIC values in control samples from the ^13^CH_4_ AOM experiments**

For the long-term AOM experiments, N_2_ headspace filled controls were prepared to show the depletion of δ^13^C-DIC (Figure S11) in contrast to the enrichment of δ^13^C-DIC observed during AOM (Figure 4A). Results presented in Figure S11 serve as negative control showing that the increase in δ^13^C-DIC observed in Figure 4A is due to the addition of ^13^CH_4_ to the incubations.


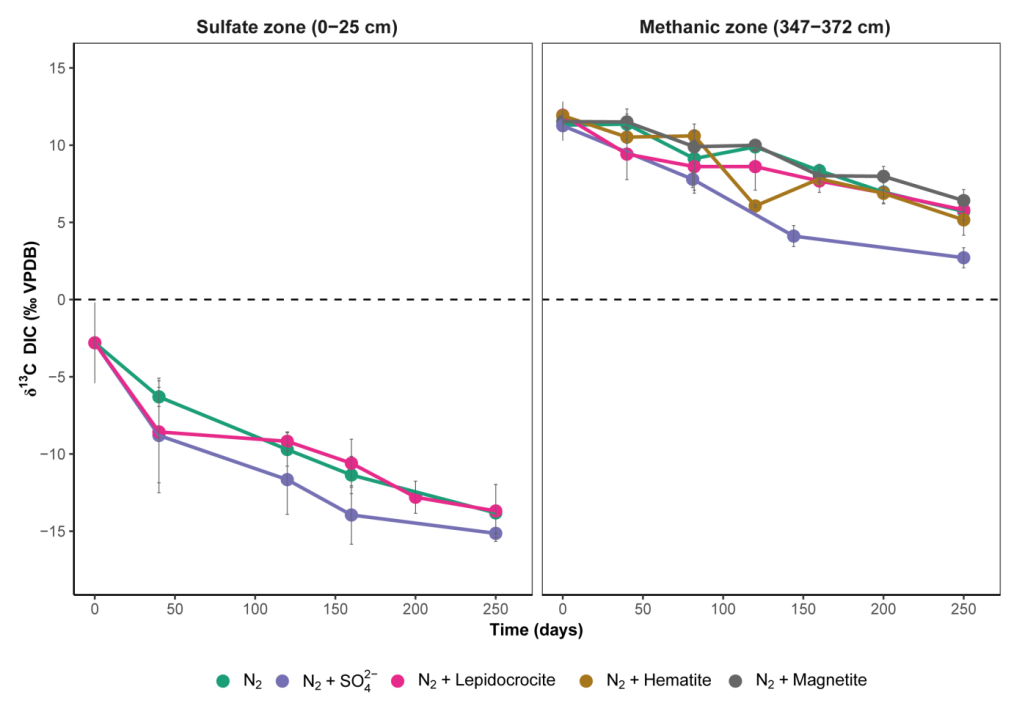


**Figure S11.** DIC measurements in control incubations over time showing the ^13^C depletion of DIC in N_2_ headspace filled controls.

**Table S1.** Maximum dissolved iron (Fe^2+^) concentrations in methanic zones of shallow coastal sediment and deep continental marine sediments across the world as shown in Figure 1.

| Location | Sediment depth (mbss) | Maximum Fe^2+^ concentrations in  the methanic zone (µM) | References |
| --- | --- | --- | --- |
| Argentine Basin | 3687 | 40 | ([Riedinger et al., 2014](#_ENREF_13)) |
| Bothnian Sea | 214 | 2000 | ([Egger et al., 2015](#_ENREF_6)) |
| Baltic Sea | 37 | 1700 | ([Egger et al., 2017](#_ENREF_4)) |
| Black Sea | 377 | 680 | ([Egger et al., 2016](#_ENREF_5)) |
| Helgoland Mud Area | 30 | 370 | ([Oni et al., 2015](#_ENREF_12)) |
| Alaskan Beaufort Sea | 280 | 80 | ([Treude et al., 2014](#_ENREF_16)) |
| Amazon Shelf | 10 | 500 | ([Aller et al., 1986](#_ENREF_1)) |
| Zambezi Fan | 1219 | 13 | ([März et al., 2008](#_ENREF_11)) |
| Aarhus Bay | 15 | 250 | ([Holmkvist et al., 2011](#_ENREF_9)) |
| Taiwan coast | 1076 | 13 | ([Lim et al., 2011](#_ENREF_10)) |
| Peru Margin | 3297 | 40 | ([D' Hondt et al., 2004](#_ENREF_3)) |
| Amazon Fan | 3511 | 130 | ([Schulz et al., 1994](#_ENREF_14)) |
| Canterbury Basin | 500 | 38 | ([Fulthorpe et al., 2011](#_ENREF_8)) |
| Bering Sea | 3173 | 60 | ([Takahashi et al., 2011](#_ENREF_15)) |

**Table S2.** Correlation analysis of dissolved Fe and Mn profile with *mcrA* gene based copies of different ANME phylotypes. Dissolved Fe and Mn concentrations (in µM), respectively, in the core HE443-010-3, were correlated to absolute gene copy numbers of each phylotype across all depths in the methanic zone. ‘n.s.’ represents non-significant p-values (> 0.05) and their confidence intervals were thereby not estimated (NA).

| ANME phylotype | Dissolved Fe | | | | Dissolved Mn | | | |
| --- | --- | --- | --- | --- | --- | --- | --- | --- |
|  | Pearson’s *r* | P(adj) | 95 % CI upper | 95% CI lower | Pearson’s *r* | P(adj) | 95 % CI upper | 95 % CI lower |
| ANME-1 | 0.28 | n.s. | NA | NA | -0.14 | n.s. | NA | NA |
| ANME-1-related | 0.64 | 0.01 | 0.23 | 0.86 | -0.67 | 0.01 | -0.87 | -0.26 |
| ANME-2a | -0.06 | n.s. | NA | NA | 0.15 | n.s. | NA | NA |
| ANME-3 | -0.08 | n.s. | NA | NA | 0.10 | n.s. | NA | NA |

**Table S3.** Development of δ^13^C values of fatty acids (in ‰) over 250 days during incubation with ^13^CH_4_ of sediment samples from the sulfate zone (S-AOM) and the methanic zone (Fe-AOM) with the latter supplemented with lepidocrocite (for details see methods). PA: phytanic acid, n.d.: not determined due to low concentration.

| S-AOM Time (days) | C_14:0_ | iC_15:0_ | aiC_15:0_ | C_15:0_ | iC_16:0_ | C_16:1ω7c_ | C_16:1ω7t_ | C_16:1ω5c_ | C_16_ | 10-Me C_16:0_ | iC_17:0_ | aiC_17:0_ | C_17:1ω6c_ | C_17:0_ | PA | C_18:1ω9c_ | C_18:1ω7c_ | C_18:0_ |
| --- | --- | --- | --- | --- | --- | --- | --- | --- | --- | --- | --- | --- | --- | --- | --- | --- | --- | --- |
| 0 | -28.0 | -29.2 | -34.5 | -24.8 | -32.0 | -6.4 | -34.2 | -58.2 | -27.3 | -29.1 | -21.8 | -32.8 | n.d. | -27.3 | -22.4 | -29.9 | -25.1 | -28.9 |
| 40 | -25.4 | -23.2 | -30.3 | -22.4 | -30.4 | 70.2 | 39.3 | -29.8 | -19.2 | -28.7 | -26.2 | -31.8 | n.d. | -24.7 | -22.6 | -24.4 | -3.4 | -28.2 |
| 82 | -20.9 | -18.5 | -23.9 | -18.2 | -25.6 | 121 | 87.2 | 25.0 | -14.0 | n.d. | n.d. | n.d. | n.d. | -27.6 | -21.7 | -18.1 | 4.0 | -27.5 |
| 120 | -20.4 | -17.4 | -25.3 | -9.1 | -23.9 | 155 | 128 | 27.2 | -6.9 | n.d. | -24.7 | -26.4 | n.d. | -25.4 | -22.2 | -14.3 | 15.0 | -24.5 |
| 160 | -16.8 | -13.2 | -9.9 | -12.9 | -19.8 | 166 | 141 | 63.4 | -7.7 | n.d. | n.d. | n.d. | n.d. | -27.4 | -22.9 | -4.5 | 16.3 | -27.3 |
| 200 | -21.9 | -15.5 | -13.1 | -23.4 | -11.7 | 121 | 80.4 | 30.6 | -19.4 | n.d. | -22.6 | -14.7 | n.d. | -31.1 | -25.0 | -16.9 | 17.4 | -27.8 |
| 250 | 1.1 | 10.2 | -11.5 | 5.2 | -10.6 | 197 | 169 | 49.6 | 32.0 | 334 | -27.2 | -9.3 | n.d. | -29.0 | -22.5 | -7.5 | 72.0 | -28.2 |
|  |  |  |  |  |  |  |  |  |  |  |  |  |  |  |  |  |  |  |
|  |  |  |  |  |  |  |  |  |  |  |  |  |  |  |  |  |  |  |
|  |  |  |  |  |  |  |  |  |  |  |  |  |  |  |  |  |  |  |
| Fe-AOM Time (days) | C_14:0_ | iC_15:0_ | aiC_15:0_ | C_15:0_ | iC_16:0_ | C_16:1ω7c_ | C_16:1ω7t_ | C_16:1ω5c_ | C_16_ | 10-Me C_16:0_ | iC_17:0_ | aiC_17:0_ | C_17:1ω6c_ | C_17:0_ | PA | C_18:1ω9c_ | C_18:1ω7c_ | C_18:0_ |
| 0 | -27.7 | -27.5 | -30.3 | -24.3 | -33.5 | -19.9 | -33.4 | -38.8 | -28.4 | n.d. | -30.6 | -31.9 | n.d. | -31.2 | -21.5 | -29.4 | -31.3 | -30.1 |
| 40 | -26.0 | -23.7 | -27.4 | -23.6 | -30.8 | 19.8 | 6.5 | -18.9 | -18.4 | n.d. | -34.5 | -37.4 | n.d. | -29.4 | -21.3 | -22.5 | -1.0 | -29.5 |
| 82 | -27.6 | -26.2 | -29.3 | -24.6 | -26.0 | -6.0 | -21.0 | -25.7 | -24.9 | -12.1 | n.d. | n.d. | n.d. | -29.2 | -23.3 | -23.6 | -22.6 | -29.2 |
| 120 | -27.6 | -25.7 | -29.1 | -24.3 | -24.1 | -8.0 | -25.5 | -33.6 | -25.1 | -19.4 | -29.7 | -32.1 | -14.5 | -31.6 | -20.0 | -27.7 | -19.9 | -30.5 |
| 160 | -27.5 | -25.7 | -28.9 | -24.8 | -24.9 | -8.6 | -32.5 | -25.6 | -26.1 | -21.8 | n.d. | n.d. | -7.0 | -30.6 | -23.1 | -24.6 | -23.7 | -29.2 |
| 200 | -24.6 | -24.2 | -27.2 | -18.7 | -29.5 | 2.6 | -13.2 | -15.0 | -20.9 | 24.3 | -34.3 | -34.0 | 47.5 | -32.5 | -22.5 | -20.8 | -19.6 | -29.5 |
| 250 | -23.1 | -24.1 | -27.5 | -10.1 | -27.5 | 9.4 | -9.2 | 7.6 | -13.1 | 27.3 | -22.6 | -26.2 | 66.0 | -23.7 | -23.7 | -4.1 | -11.3 | -30.1 |

**Table S4.** Total uptake of ^13^CH_4_ into bacterial fatty acids (in ng ^13^C/g dw) after 250 days based on the excess of ^13^C relative to the original sediment (T_0_ sample) during incubation of sediment samples from the sulfate zone (S-AOM) and the methanic zone (Fe-AOM) with the latter supplemented with lepidocrocite (for details see methods). Negative ^13^C-uptake of fatty acids because of absence of increase in δ^13^C values over the incubation period is considered zero. n.d.: not determined due to low concentration. *: Fatty acids with no δ^13^C value in the T_0_ sample. To calculate ^13^C-uptake for these, the average δ^13^C value of all other fatty acids is used, i.e. -30‰.

|  | C_14:0_ | iC_15:0_ | aiC_15:0_ | C_15:0_ | iC_16:0_ | C_16:1ω7c_ | C_16:1ω7t_ | C_16:1ω5c_ | C_16_ | 10-Me C_16:0_ | iC_17:0_ | aiC_17:0_ | C_17:1ω6c_ | C_17:0_ | C_18:1ω9c_ | C_18:1ω7c_ | C_18:0_ |
| --- | --- | --- | --- | --- | --- | --- | --- | --- | --- | --- | --- | --- | --- | --- | --- | --- | --- |
| S-AOM | 0.08 | 0.04 | 0.04 | 0.03 | 0.02 | 0.27 | 0.07 | 0.05 | 1.06 | 0.59 | 0 | 0.01 | n.d. | 0 | 0.03 | 0.10 | 0.01 |
| Fe-AOM | 0.03 | 0.01 | 0.01 | 0.03 | 0.01 | 0.11 | 0.02 | 0.02 | 0.50 | 0.05* | 0.01 | 0.00 | 0.11* | 0.01 | 0.06 | 0.03 | 0.00 |

**Table S5.** Protein BLAST analysis of sequences from the *pmoA* cloning experiments.

| **Clone name** | **Incubation setup** | **Sequence length (bp)** | **Translated protein length without stop codons (amino acids)** | **Protein BLAST hit(s)** | **Max  score** | **Total  score** | **Query  coverage (%)** | **E  value** | **Indentity  (%)** | **Accession** |
| --- | --- | --- | --- | --- | --- | --- | --- | --- | --- | --- |
| P5 | ^13^CH_4_ + Molybdate + Magnetite | 355 | 97 | APS reductase alpha subunit [Endosymbiont of Sclerolinum contortum] | 50.4 | 50.4 | 78 | 1.0E-05 | 37 | CAP03145 |
|  |  |  |  | Carbon monoxide dehydrogenase activity/acetyl-CoA synthase, partial [Terrisporobacter glycolicus] | 49.7 | 49.7 | 23 | 9.0E-05 | 96 | CCA62924 |
|  |  |  |  | Flavin oxidoreductase [Stenotrophomonas chelatiphaga] | 48.1 | 48.1 | 21 | 3.0E-04 | 100 | CDQ30636 |
|  |  |  |  | Nitrite reductase [uncultured bacterium] | 45.8 | 45.8 | 26 | 7.0E-04 | 82 | BAH95949 |
|  |  |  |  | Methyl-conenzyme M reductase alpha subunit [uncultured archaeon] | 45.8 | 45.8 | 23 | 0.001 | 88 | CBF64715 |
|  |  |  |  | Putative heat shock protein isoform a [Lithodes maja] | 44.7 | 44.7 | 22 | 0.003 | 92 | CEK41093 |
|  |  |  |  | Cytochrome P450 CYP153 alkane hydroxylase [uncultured bacterium] | 35.8 | 35.8 | 34 | 6.3 | 66 | CCO96973 |
| P1 | ^13^CH_4_ + Molybdate + Magnetite | 333 | 110 | No significant similarity found | - | - | - | - | - | - |
| P6 | ^13^CH_4_ + Molybdate + Magnetite | 333 | 110 | No significant similarity found | - | - | - | - | - | - |
| P9 | ^13^CH_4_ + Molybdate + Magnetite | 333 | 110 | No significant similarity found | - | - | - | - | - | - |
| L1 | ^13^CH_4_ + Molybdate + Lepidocrocite | 332 | 110 | No significant similarity found | - | - | - | - | - | - |
| L3 | ^13^CH_4_ + Molybdate + Lepidocrocite | 333 | 110 | No significant similarity found | - | - | - | - | - | - |
| L4 | ^13^CH_4_ + Molybdate + Lepidocrocite | 333 | 110 | No significant similarity found | - | - | - | - | - | - |
| L6 | ^13^CH_4_ + Molybdate + Lepidocrocite | 333 | 110 | No significant similarity found | - | - | - | - | - | - |
| L7 | ^13^CH_4_ + Molybdate + Lepidocrocite | 333 | 109 | No significant similarity found | - | - | - | - | - | - |
| L8 | ^13^CH_4_ + Molybdate + Lepidocrocite | 333 | 110 | No significant similarity found | - | - | - | - | - | - |

**Table S6.** δ^13^C values (in ‰) of phytanes (phy) and biphytanes (bphy) derived from archaeal membrane intact polar lipids (IPLs) over 250 days of incubation with ^13^CH_4_ of sediment samples from the sulfate zone (S-AOM) and the methanic zone (Fe-AOM) with the latter supplemented with lepidocrocite (for details see methods). n.d.: not determined due to low concentration.

| S-AOM Time (days) | phy | bphy 0 | bphy 1 | bphy 2 | bphy 3 |
| --- | --- | --- | --- | --- | --- |
| 0 | n.d. | -28.4 | n.d. | -23.5 | -18.2 |
| 40 | n.d. | -30.9 | n.d. | -22.0 | -18.6 |
| 82 | n.d. | -29.3 | n.d. | -23.2 | -18.5 |
| 120 | n.d. | -30.8 | n.d. | -24.2 | -18.1 |
| 160 | n.d. | -31.3 | n.d. | -24.1 | -18.2 |
| 200 | n.d. | -30.3 | n.d. | -24.6 | -18.9 |
| 250 | n.d. | -30.8 | n.d. | -24.3 | -19.6 |
|  |  |  |  |  |  |
|  |  |  |  |  |  |
|  |  |  |  |  |  |
| Fe-AOM Time (days) | phy | bphy 0 | bphy 1 | bphy 2 | bphy 3 |
| 0 | -32.0 | -36.0 | n.d. | -28.7 | -19.1 |
| 40 | -32.2 | -35.2 | n.d. | -29.4 | -17.3 |
| 82 | -32.3 | -34.9 | n.d. | -27.9 | -17.7 |
| 120 | -33.3 | -34.9 | n.d. | -28.7 | -19.1 |
| 160 | -33.3 | -34.7 | n.d. | -26.5 | -18.2 |
| 200 | -31.8 | -34.7 | n.d. | -25.8 | -19.6 |
| 250 | -31.9 | -34.9 | n.d. | -29.8 | -18.0 |

**Table S7.** Information on iron oxides used in this study.

| **Iron oxide** | | **Predominant particle size (µm)** | **Iron oxide content (%)** | **Particle shape** |
| --- | --- | --- | --- | --- |
| Bayferrox 11 | Hematite | 0.09 | >99.1 | Spherical |
| Bayferrox 943 | Lepidocrocite | 0.05 x 0.3 | >99.4 | Acicular |
| Bayoxide E 8709 | Magnetite | 0.2 | >99.5 |  |

**Table S8.** Back-of-the-envelope calculation: **Could Fe-AOM rates in marine sediments account for the oxidation of the entire estimated diffusive flux of methane into the SMT?** Since inner shelf sediments (0-50 m water depths) have the highest sedimentation rate among all ocean sediments, unreacted Fe oxides may be abundantly present in the inner shelf sediments below the SMT and might be available for Fe-AOM The following assumptions were made: Seafloor area of the inner shelf (0-50 m) is 1.18 x 10^7^ km^2^ and diffusive methane flux for the inner shelf sediments is 2.4 T mol yr^-1^ ([Egger et al., 2018](#_ENREF_7)). Using Fe-AOM rates from this and other studies, we calculated the volume of sediment required to achieve the oxidation of the diffusive methane flux in the SMT, the corresponding area and depth of inner shelf sediments required. The estimates show that sediment Fe-AOM rates could account for oxidizing the entire methane flux in the surface (3 sites). However, methanic sediment AOM rates are for the most part too low (except Helgoland Mud Area) requiring a magnitude of sediment of up to 14 km, which is not available. Since S-AOM is in place, Fe-AOM can be much lower and still contribute to AOM in marine sediments; more data points are required to realistically estimate Fe-AOM rates. ^a^ represents rates measured from ^14^CH_4_ incubations, ^b^ represents rates estimated using geochemical modelling studies, and ^c^ represents rates measured from ^13^CH_4_ incubations.

| **Site** | **Sediment  zone** | **Fe-AOM rates  (mol CH_4_ cm^-3^ yr^-1^)** | **Sediment  volume  (km^3^)** | **Sediment  area  (km^2^)** | **Sediment depth (m)** | **References for Fe-AOM rates** |
| --- | --- | --- | --- | --- | --- | --- |
| North Sea | **Methanic** | 3.47E-08 ^a^ | 6.92E+04 | 6.92E+07 | 5.88 | This study |
| Baltic Sea |  | 1.10E-09 ^b^ | 2.18E+06 | 2.18E+09 | 185.37 | ([Egger et al., 2017](#_ENREF_4)) |
| Black Sea |  | 1.46E-11 ^b^ | 1.64E+08 | 1.64E+11 | 13975.89 | ([Egger et al., 2016](#_ENREF_5)) |
|  |  |  |  |  |  |  |
| Bothnian Sea | **Surface** | 1.30E-06 ^c^ | 1.85E+03 | 1.85E+06 | 0.16 | ([Egger et al., 2015](#_ENREF_6)) |
| Eel River Basin seep |  | 6.00E-06 ^c^ | 4.00E+02 | 4.00E+05 | 0.03 | ([Beal et al., 2009](#_ENREF_2)) |
| Chowder Hill hydrothermal vent |  | 5.90E-05 ^a^ | 4.07E+01 | 4.07E+04 | 0 | ([Wankel et al., 2012](#_ENREF_17)) |

**References**

Aller, R.C., Mackin, J.E., and Cox, R.T. (1986). Diagenesis of Fe and S in Amazon inner shelf muds: apparent dominance of Fe reduction and implications for the genesis of ironstones. *Cont. Shelf. Res.* 6**,** 263-289.

Beal, E.J., House, C.H., and Orphan, V.J. (2009). Manganese- and iron-dependent marine methane oxidation. *Science* 325**,** 184-187.

D' Hondt, S., Jørgensen, B.B., Miller, D.J., Batzke, A., Blake, R., Cragg, B.A., Cypionka, H., Dickens, G.R., Ferdelman, T., Hinrichs, K.-U., Holm, N.G., Mitterer, R., Spivack, A., Wang, G., Bekins, B., Engelen, B., Ford, K., Gettemy, G., Rutherford, S.D., Sass, H., Skilbeck, C.G., Aiello, I.W., Guèrin, G., House, C.H., Inagaki, F., Meister, P., Naehr, T., Niitsuma, S., Parkes, R.J., Schippers, A., Smith, D.C., Teske, A., Wiegel, J., Padilla, C.N., and Acosta, J.L.S. (2004). Distributions of microbial activities in deep subseafloor sediments. *Science* 306**,** 2216-2221.

Egger, M., Hagens, M., Sapart, C.J., Dijkstra, N., Van Helmond, N.a.G.M., Mogollón, J.M., Risgaard-Petersen, N., Van Der Veen, C., Kasten, S., Riedinger, N., Böttcher, M.E., Röckmann, T., Jørgensen, B.B., and Slomp, C.P. (2017). Iron oxide reduction in methane-rich deep Baltic Sea sediments. *Geochim. Cosmochim. Acta* 207**,** 256-276.

Egger, M., Kraal, P., Jilbert, T., Sulu-Gambari, F., Sapart, C.J., Röckmann, T., and Slomp, C.P. (2016). Anaerobic oxidation of methane alters sediment records of sulfur, iron and phosphorus in the Black Sea. *Biogeosciences* 13**,** 5333-5355.

Egger, M., Rasigraf, O., Sapart, C.J., Jilbert, T., Jetten, M.S.M., Röckmann, T., Van Der Veen, C., Bândă, N., Kartal, B., Ettwig, K.F., and Slomp, C.P. (2015). Iron-mediated anaerobic oxidation of methane in brackish coastal sediments. *Environ. Sci. Technol.* 49**,** 277-283.

Egger, M., Riedinger, N., Mogollón, J.M., and Jørgensen, B.B. (2018). Global diffusive fluxes of methane in marine sediments. *Nature Geosci* 11**,** 421-425.

Fulthorpe, C.S., Hoyanagi, K., Blum, P., and Scientists, I.E. (2011). IODP Expedition 317: exploring the record of sea-level change off New Zealand. *Sci. Dril.* 12**,** 4-14.

Holmkvist, L., Ferdelman, T.G., and Jørgensen, B.B. (2011). A cryptic sulfur cycle driven by iron in the methane zone of marine sediment (Aarhus Bay, Denmark). *Geochim. Cosmochim. Acta* 75**,** 3581-3599.

Lim, Y.C., Lin, S., Yang, T.F., Chen, Y.-G., and Liu, C.-S. (2011). Variations of methane induced pyrite formation in the accretionary wedge sediments offshore southwestern Taiwan. *Mar. Petrol. Geol.* 28**,** 1829-1837.

März, C., Hoffmann, J., Bleil, U., De Lange, G.J., and Kasten, S. (2008). Diagenetic changes of magnetic and geochemical signals by anaerobic methane oxidation in sediments of the Zambezi deep-sea fan (SW Indian Ocean). *Mar. Geol.* 255**,** 118-130.

Oni, O., Miyatake, T., Kasten, S., Richter-Heitmann, T., Fischer, D., Wagenknecht, L., Ksenofontov, V., Kulkarni, A., Blumers, M., Shylin, S., Costa, B., Klingelhöfer, G., and Friedrich, M. (2015). Distinct microbial populations are tightly linked to the profile of dissolved iron in the methanic sediments of the Helgoland mud area, North Sea. *Front. Microbiol.* 6.

Riedinger, N., Formolo, M.J., Lyons, T.W., Henkel, S., Beck, A., and Kasten, S. (2014). An inorganic geochemical argument for coupled anaerobic oxidation of methane and iron reduction in marine sediments. *Geobiology* 12**,** 172-181.

Schulz, H.D., Dahmke, A., Schinzel, U., Wallmann, K., and Zabel, M. (1994). Early diagenetic processes, fluxes, and reaction rates in sediments of the South Atlantic. *Geochim. Cosmochim. Acta* 58**,** 2041-2060.

Takahashi, K., Ravelo, A.C., Alvarez Zarikian, C., and The, I.E.S. (2011). IODP Expedition 323- Pliocene and pleistocene paleoceanographic changes in the Bering Sea. *Sci. Dril.* 11**,** 4-13.

Treude, T., Krause, S., Maltby, J., Dale, A.W., Coffin, R., and Hamdan, L.J. (2014). Sulfate reduction and methane oxidation activity below the sulfate-methane transition zone in Alaskan Beaufort Sea continental margin sediments: implications for deep sulfur cycling. *Geochim. Cosmochim. Acta* 144**,** 217-237.

Wankel, S.D., Adams, M.M., Johnston, D.T., Hansel, C.M., Joye, S.B., and Girguis, P.R. (2012). Anaerobic methane oxidation in metalliferous hydrothermal sediments: influence on carbon flux and decoupling from sulfate reduction. *Environ. Microbiol.* 14**,** 2726-2740.
